# Microbial activity response to hydrogen injection in thermophilic anaerobic digesters revealed by genome-centric metatranscriptomics

**DOI:** 10.1186/s40168-018-0583-4

**Published:** 2018-10-27

**Authors:** Alessandra Fontana, Panagiotis G. Kougias, Laura Treu, Adam Kovalovszki, Giorgio Valle, Fabrizio Cappa, Lorenzo Morelli, Irini Angelidaki, Stefano Campanaro

**Affiliations:** 1 Department for Sustainable Food Process, DiSTAS, Catholic University of the Sacred Heart, 29122 Piacenza, Italy; 2 Department of Environmental Engineering, Technical University of Denmark, 2800 Kongens Lyngby, Denmark; 3 Department of Biology, University of Padua, 35131 Padua, Italy

**Keywords:** Anaerobic digestion, CO_2_ fixation, Biomethane, Metagenomics, Metatranscriptomics, Metagenome assembled genomes, Cheese wastes, Microbial community, Short-chain fatty acids, Syntrophism

## Abstract

**Background:**

The expansion of renewable energy produced by windmills and photovoltaic panels has generated a considerable electricity surplus, which can be utilized in water electrolysis systems for hydrogen production. The resulting hydrogen can then be funneled to anaerobic digesters for biogas upgrading (biomethanation) purposes (power-to-methane) or to produce high value-added compounds such as short-chain fatty acids (power-to-chemicals).

Genome-centric metagenomics and metatranscriptomic analyses were performed to better understand the metabolic dynamics associated with H_2_ injection in two different configurations of anaerobic digesters treating acidic wastes, specifically cheese manufacturing byproducts. These approaches revealed the key-genes involved in methanation and carbon fixation pathways at species level.

**Results:**

The biogas upgrading process in the single-stage configuration increased the CH_4_ content by 7%. The dominant methanogenic species responsible for the upregulation of the hydrogenotrophic pathway in this reactor was *Methanothermobacter wolfeii* UC0008. In the two-stage configuration, H_2_ injection induced an upregulation of CO_2_ fixation pathways producing short-chain fatty acids, mainly acetate and butyrate. In this configuration, the abundant species *Anaerobaculum hydrogeniformans* UC0046 and *Defluviitoga tunisiensis* UC0050 primarily upregulated genes related to electron transport chains, suggesting putative syntrophisms with hydrogen scavenger microbes. Interestingly, *Tepidanaerobacter acetatoxydans* UC0018 did not act as an acetate-oxidizer in either reactor configurations, and instead regulated pathways involved in acetate production and uptake. A putative syntrophic association between *Coprothermobacter proteolyticus* UC0011 and *M*. *wolfeii* UC0008 was proposed in the two-stage reactor. In order to support the transcriptomic findings regarding the hydrogen utilization routes, an advanced bioconversion model was adapted for the simulation of the single- and two-stage reactor setups.

**Conclusions:**

This is the first study investigating biogas reactor metatranscriptome dynamics following hydrogen injection for biomethanation and carbon fixation to short-chain fatty acids purposes. The same microbes showed different patterns of metabolic regulation in the two reactor configurations. It was observed an effect of the specialized acidogenic reactor on the overall microbial consortium composition and activity in the two-stage digester. There were also suggested the main species responsible for methanation, short-chain fatty acids production, and electron transport chain mechanisms, in both reactor configurations.

**Electronic supplementary material:**

The online version of this article (10.1186/s40168-018-0583-4) contains supplementary material, which is available to authorized users.

## Background

The increasing demand for renewable energy sources (RES) promoted the expanded use of windmills and photovoltaic panels. The installed global wind capacity increased by 10.8% in 2017, with China and the USA as the major producers of electricity from wind [1]. This expansion has led to the production of a considerable electricity surplus, which cannot be easily stored in batteries due to high cost or injected into the national grid, since it could cause electrical instabilities. This electricity surplus could be used for H_2_ production via water electrolysis [2]. Nevertheless, hydrogen is highly volatile and therefore difficult to store and transport, and is associated with some environmental risks. Alternatively, this H_2_ could be used for biogas upgrading purposes (power-to-methane) or for the production of high value-added compounds such as fatty acids (mainly short-chain carboxylates) and alcohols (power-to-chemicals), via anaerobic digestion (AD), generating an energy gain in the form of methane along with organic waste valorization [3–6].

The process of anaerobic digestion relies on a complex microbial syntrophic chain, which degrades the organic matter into various byproducts such as short-chain fatty acids (SCFAs) and eventually to biogas (i.e., methane and carbon dioxide). Different operating conditions can direct the process toward higher CH_4_ or SCFAs yields. Specifically, pH, temperature, and hydraulic retention time (HRT) represent the most important factors in SCFAs accumulation [7]. SCFAs, also known as volatile fatty acids (VFAs), consist of six or fewer carbon atoms and can be used in the synthesis of a wide range of compounds, including biosurfactants, bioflocculants, and bioplastics (polyhydroxyalkanoates (PHAs)) [8, 9].

SCFAs are among the main products of cheese whey AD and, despite the low alkalinity typical of this waste, Fontana et al. have recently demonstrated the higher efficiency of a two-stage continuous stirred tank reactor (CSTR) than a single-stage configuration for treating whey permeate and hard-cheese solid waste to produce biogas [10]. However, the different reactor configurations evaluated in the study also indicated the potential to use cheese wastes AD for carbon dioxide fixation to SCFAs, such as butyrate and acetate.

The conversion of these chemical compounds is performed by an intricate set of microbes where different species cooperate or compete to generate the final product. Functional properties of individual species can be explored by reconstructing their genomes from the metagenome. This metagenomic approach allows the identification of the so-called metagenome assembled genomes (MAGs), which can be successfully reconstructed by binning the scaffolds that were previously assembled from the shotgun reads. Among the existing binning strategies [11, 12], Campanaro et al. developed a specific methodology that was applied to characterize microbial communities in biogas reactors [13]. This genome-centric approach, combined with the metatranscriptomics, provides a powerful method to uncover the phylogenetic and metabolic properties in anaerobic digestion without relying on culture-dependent techniques. Particularly, the possibility to align the RNA-seq reads to the MAGs allows to investigate the activity of specific microbes and to monitor the modifications occurring during the alteration of the environmental conditions.

The current study aimed to investigate the effect of exogenous H_2_ injection on microbial activity in two different thermophilic reactor configurations (single and two-stage continuous stirred tank reactors) treating cheese wastes. Particularly, improvements in biogas methanation and/or CO_2_ fixation to SCFAs by CO_2_ reduction pathways have been evaluated. Genome-centric metagenomics and metatranscriptomics allowed an in-depth analysis of differential gene expression following H_2_ addition by the most abundant MAGs. The outcomes of the present study also reveal putative syntrophic associations between key microbial groups and methanogenic archaea.

## Methods

### Biogas reactors’ configuration

**Fig. 1:**
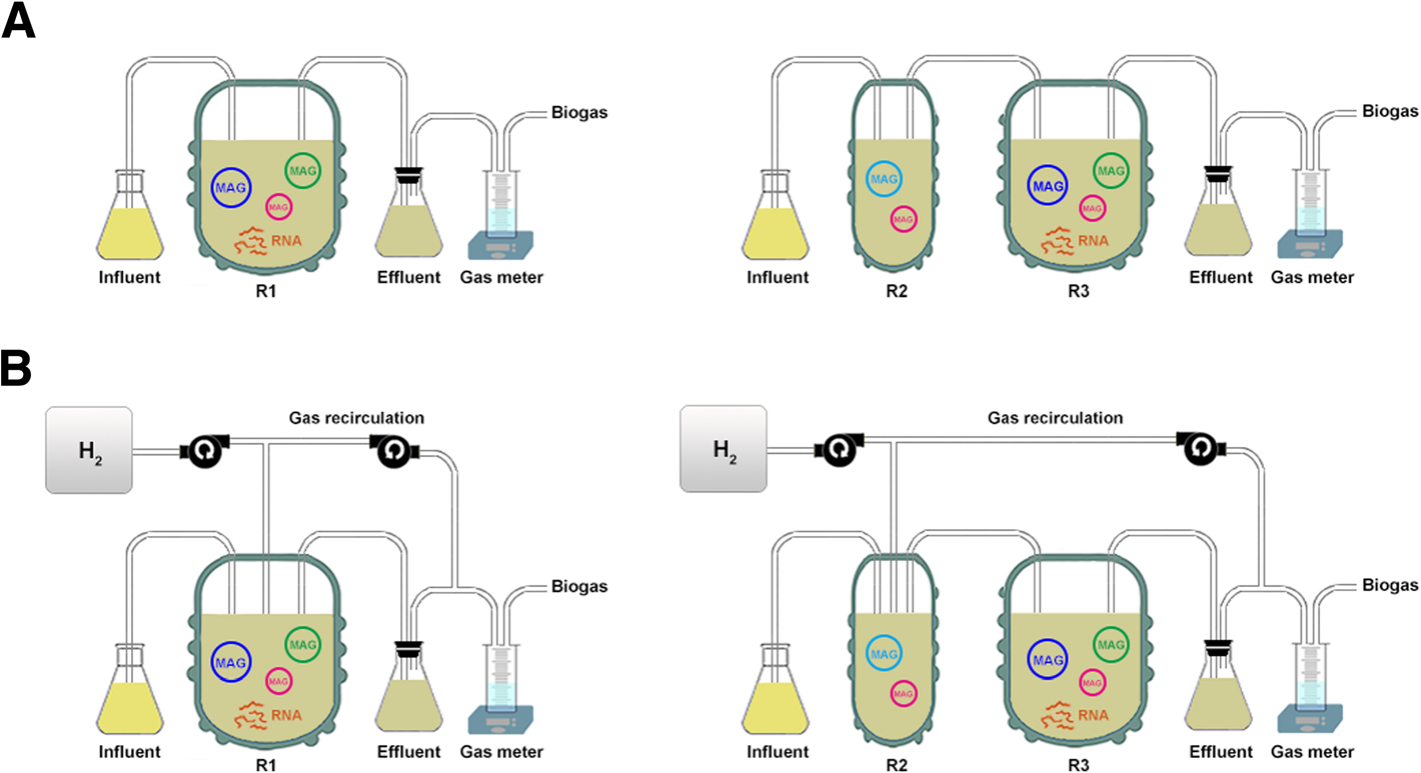
Experimental set up in phase I, before H_2_ injection (**a**) and phase II, after H_2_ injection (**b**). The abundance shifts of the species (MAGs) and the differential gene expression between the two phases were evaluated. *MAG* metagenome assembled genome, *R1* single-stage reactor, *R2* acidogenic reactor of the two-stage, *R3* methanogenic reactor of the two-stage

The experiment was carried out in a single and two-stage CSTRs, denoted as R1 and R2–R3, respectively, as described by Fontana et al. [10]. The reactors had a total working volume of 3 L; they were continuously stirred at 150 rpm using magnetic stirrers and equipped with thermal jackets to maintain the operating temperature at 55 ± 1 °C. Initially, all reactors were inoculated with thermophilic inoculum obtained from Snertinge biogas plant, Denmark. The experiment was divided in two phases: phase I, when the reactors were fed exclusively with cheese whey permeate and cheese waste powder, and phase II, when exogenous H_2_ was added to the reactors (Fig. 1). The H_2_ gas was provided in the reactors R1 and R2 by using a peristaltic pump operating at a flow rate of 1.7 mL/min and was injected through Al_2_O_3_ ceramic membranes having pore size of 1.2 μm. For the two-stage CSTR system, the output gas and the liquid effluent from the acidogenic reactor (R2) was transferred pneumatically to the methanogenic reactor (R3) via a connecting tube (Fig. 1). In order to maximize the gas-liquid mass transfer, another peristaltic pump was connected with each reactor to recirculate the output gas. The recirculation flow rate was set to 0.7 mL/L min. During both experimental phases, the organic loading rate (OLR) was set at 2.4 g COD/L day, and thus, the hydraulic retention time (HRT) was maintained at 15 days (split in 3 and 12 days for R2 and R3, respectively). The characteristics of the feedstock are described in Fontana et al. [10]. The influent feedstock was automatically provided four times per day using controlled peristaltic pumps. Sodium hydroxide addition in R1 and R3 was applied whenever the pH dropped below 6.5. The H_2_ flow rate was defined in relation to the stoichiometry of hydrogenotrophic methanogenesis reaction (4 H_2_/1 CO_2_ mol/mol) as described by Bassani et al. [14]. Half H_2_ volume (~ 820 mL/L day) was injected to gradually adapt the system to the new condition. Mass balance calculations were carried out as indicated in the Additional file 1.

The H_2_ utilization efficiency was calculated as previously described [15], using the following Eq. (1):

1 $$ {H}_2\ \mathrm{utilization}\ \mathrm{efficiency}=\frac{{\mathrm{H}}_2\ \mathrm{in}\mathrm{jected}\ \left(\raisebox{1ex}{$\mathrm{mL}$}\!\left/ \!\raisebox{-1ex}{$\mathrm{L}\bullet \mathrm{day}$}\right.\right)-{\mathrm{H}}_2\kern0.5em \mathrm{in}\ \mathrm{biogas}\ \left(\raisebox{1ex}{$\mathrm{mL}$}\!\left/ \!\raisebox{-1ex}{$\mathrm{L}\bullet \mathrm{day}$}\right.\right)}{{\mathrm{H}}_2\ \mathrm{in}\mathrm{jected}\ \left(\raisebox{1ex}{$\mathrm{mL}$}\!\left/ \!\raisebox{-1ex}{$\mathrm{L}\bullet \mathrm{day}$}\right.\right)}\times 100 $$

The CO_2_ conversion efficiency was calculated as follows (2):

2 $$ {\mathrm{CO}}_2\ \mathrm{conversion}\ \mathrm{efficiency}=\frac{\overline{{\mathrm{CO}}_2}\ \mathrm{phaseI}\ \left(\raisebox{1ex}{$\mathrm{mL}$}\!\left/ \!\raisebox{-1ex}{$\mathrm{L}\bullet \mathrm{day}$}\right.\right)-{\mathrm{CO}}_2\kern0.5em \mathrm{in}\ \mathrm{biogas}\ \mathrm{phase}\ \mathrm{II}\ \left(\raisebox{1ex}{$\mathrm{mL}$}\!\left/ \!\raisebox{-1ex}{$\mathrm{L}\bullet \mathrm{day}$}\right.\right)}{\overline{{\mathrm{CO}}_2}\ \mathrm{phaseI}\ \left(\raisebox{1ex}{$\mathrm{mL}$}\!\left/ \!\raisebox{-1ex}{$\mathrm{L}\bullet \mathrm{day}$}\right.\right)}\times 100 $$

### Sample collection

For metatranscriptomic analyses, triplicate samples (~ 30 mL each) were collected from R1 and R3 at steady-state reactor operation of phase I (i.e., period with stable biogas production with a daily variation lower than 10% for at least 5 days), and after 1 week from the H_2_ injection (phase II). Replicates obtained from phase I were indicated as R1_a, R1_b, R1_c, R3_a, R3_b, R3_c, while replicates from phase II were denoted as R1H_a, R1H_b, R1H_c, R3H_a, R3H_b, R3H_c.

### DNA and RNA extraction and sequencing

Samples were centrifuged at 10,000 rpm for 10 min and the supernatant was discarded leaving ~ 1 g of pellet. To avoid RNA degradation, 3.5 mL of phenol/chloroform (pH 6.7/8.0) premixed with isoamyl alcohol (25:24:1) (Amresco, Incorporated) was added to the pellet after centrifugation and the samples were immediately processed for RNA extraction, as previously reported [16]. Total RNA was extracted with the RNA PowerSoil® Kit (MO BIO laboratories, Carlsbad, CA, USA). RNA integrity was checked using Agilent Bioanalyzer. The quality of the samples (RIN > 6) obtained in previous studies [16] was similar and fulfilled the criteria for library preparation and RNA sequencing of the Ramaciotti Centre for Genomics. RNA libraries were prepared from individual samples using the TruSeq RNA Library Preparation Kit (Illumina, San Diego, CA). All the RNA samples were sequenced single-end (75 bp) using NextSeq 500 system (Illumina, San Diego, CA). Reads in FASTQ format were quality-filtered and the adaptors were removed using Trimmomatic software (v0.33) [17] with the following parameters: ILLUMINACLIP:NexteraSE-SE:2:30:10 LEADING:10 TRAILING:10 SLIDINGWINDOW:4:15 MINLEN:50. From 17,754,036 to 32,203,531 sequences were obtained considering the 12 samples.

Genomic DNA was extracted from the same initial samples with the RNA PowerSoil® DNA Elution Accessory Kit (MO BIO laboratories, Carlsbad, CA, USA). Genomic DNA integrity was determined using agarose gel electrophoresis. Samples were sequenced, using NextSeq 500 sequencing technology and Nextera XT kit (Illumina, San Diego, CA) for library preparation (150 + 150 bp). The quality and the quantity of the extracted DNA and RNA were also determined using both NanoDrop (ThermoFisher Scientific, Waltham, MA, USA) and Qubit fluorometer (Life Technologies, Carlsbad, CA, USA).

Additional liquid samples (4 mL) for metagenomic analyses were collected in three replicates and in three time points (days 41, 52, and 61) at steady-state condition from R1 and R3. DNeasy PowerSoil Kit (QIAGEN, Germany) was used for genomic DNA extraction with minor modifications (purification with 1 mL of Phe:Chl:IAA pH 8, Sigma-Aldrich, DK). Microbial community composition was determined using the V3–V4 hypervariable regions of 16S rRNA gene using universal primers Pro341F and Pro805R [18]. Amplicon preparation and sequencing were performed at BMR Genomics S.r.l. (Padua, Italy) using Illumina MiSeq platform. Raw sequencing data were submitted to the sequence read archive database (SRA) of NCBI under the BioProject PRJNA490620 and the accessions SAMN10054307-SAMN10054315 for R1 samples and SAMN10054316-SAMN10054324 for R3 samples. Data analysis was performed using CLC Workbench software (V.8.0.2) with microbial genomics module plug in (QIAGEN Bioinformatics, Germany) as previously described [5] with “quality limit” parameter set at 0.01. In brief, chimeras filtering, operative taxonomical units clustering, taxonomic assignment (with Greengenes v13_5 database), and alpha and beta diversity (Unweighted UniFrac) calculation were done using standard parameters. Additionally, relative abundances of the OTUs were used to determine the beta diversity value (Whittaker method) and all the possible comparisons between replicates, time points, and reactors were performed.

### Reads alignment, gene expression calculation, and statistical analysis

Gene expression analysis was performed considering as reference the global metagenome assembly. Gene finding and annotation were reported in a previous study [10] and in brief were performed as follows: gene prediction on the entire assembly was determined with Prodigal (v2.6.2) run in metagenomic mode [19]. Protein-encoding genes were annotated using reverse-position specific BLAST algorithm and using Clusters of orthologous groups COG and Pfam database [20, 21]; only results with *e* value lower than 1e^−5^ were retained. Genes were also annotated according to Kyoto Encyclopedia of Genes and Genomes (KEGG) using GhostKOALA [22] and to EggNOG 4.5.1 using eggNOG-mapper [23]. Filtered reads were aligned on reference metagenome assembly using bowtie2 (v2.2.4) [24] and the number of reads mapped per each gene was determined from the “sam” file using HTSeq (v0.6.1) [25] with the options “-count” and “intersection-non empty.” Each gene was previously assigned to the correspondent MAG with a binning strategy previously described [10]. MAGs abundance was considered directly related to scaffold coverage, which was determined by aligning the shotgun reads on the assembly as described by Campanaro and co-workers [26]. Coverage values determined for MAGs were visualized with MeV [27]. To evaluate the changes in abundance of the main MAGs after H_2_ injection (phase II), a comparison with the coverage values of the previously described MAGs, before H_2_ injection (phase I) [10], has been carried out. The statistical analysis was performed independently for each MAG using edgeR software [28] and the differentially expressed genes were filtered considering the *p* value (pVal.Tgw < 0.05) and the coverage ratio (> 2-fold change). KEGG pathway maps were obtained with the “KEGG Mapper Search&Color Pathway” tool [29]. To identify the COG and KEGG functional classes statistically enriched of differentially expressed genes, the procedure described by Treu et al. [30] was followed. Briefly, 10,000 random samplings of *n* genes (where *n* is the number of genes for each COG or KEGG class) were performed on the entire data set of genes expressed (data set S3) using a Perl script implementing the “rand()” function. Assuming differentially expressed (DE) as the number of differentially expressed genes in a group of *n* genes, the fraction of randomly selected samples having differentially expressed genes equal to or higher than DE was calculated. If this fraction was lower than the significance level (0.05), the enrichment of genes differentially expressed in the *n* genes was considered significant. Additional statistical analyses have been performed on a selection of MAGs; Fisher’s exact test was applied to define the KEGG pathways including a significant number of differentially expressed genes. Finally, multiple test correction was performed for calculating false discovery rate (data set S4).

Canonical correspondence analysis (CCA) was performed using the R functions implemented in VEGAN v2.4-4 [31], while correspondence analysis (CA) based on Pearson calculation and non-metric multidimensional scaling (NMDS) were performed with R following the procedure described by Torondel et al. [32].

Raw sequence data have been deposited at Sequence Read Archive (SRA) under the BioProject PRJNA394669 and the accessions SAMN07367931-SAMN07367939 and SAMN07638604-SAMN07638612 for DNA pre- and post-H_2_, respectively; SAMN07367931-SAMN07367933 and SAMN07367937-SAMN07367939 for RNA pre-H_2_, SAMN07638604-SAMN07638606 and SAMN07638610-SAMN07638612 for RNA post-H_2_.

### Modeling of the experimental setup

Software simulations of the single and two-stage reactors were carried out using the complex anaerobic digestion simulation suite (BioModel) developed by Angelidaki and co-workers [33, 34], which was later extended to account for biogas upgrading experiments with hydrogen injection [35]. For improved simulation accuracy, previously optimized model parameters were taken from Kovalovszki et al. [36]. The tool was further enabled to simulate two-stage reactor configurations, through the inclusion of intermediate compounds and gases produced in the acidogenic reactor (R2) in the feed of the methanogenic reactor (R3). In addition, syntrophic acetate oxidizing microbial groups were also considered in the model, making it possible to simulate the dynamic interactions between acetoclastic and hydrogenotrophic methanogenic groups.

## Results and discussion

**Fig. 2:**
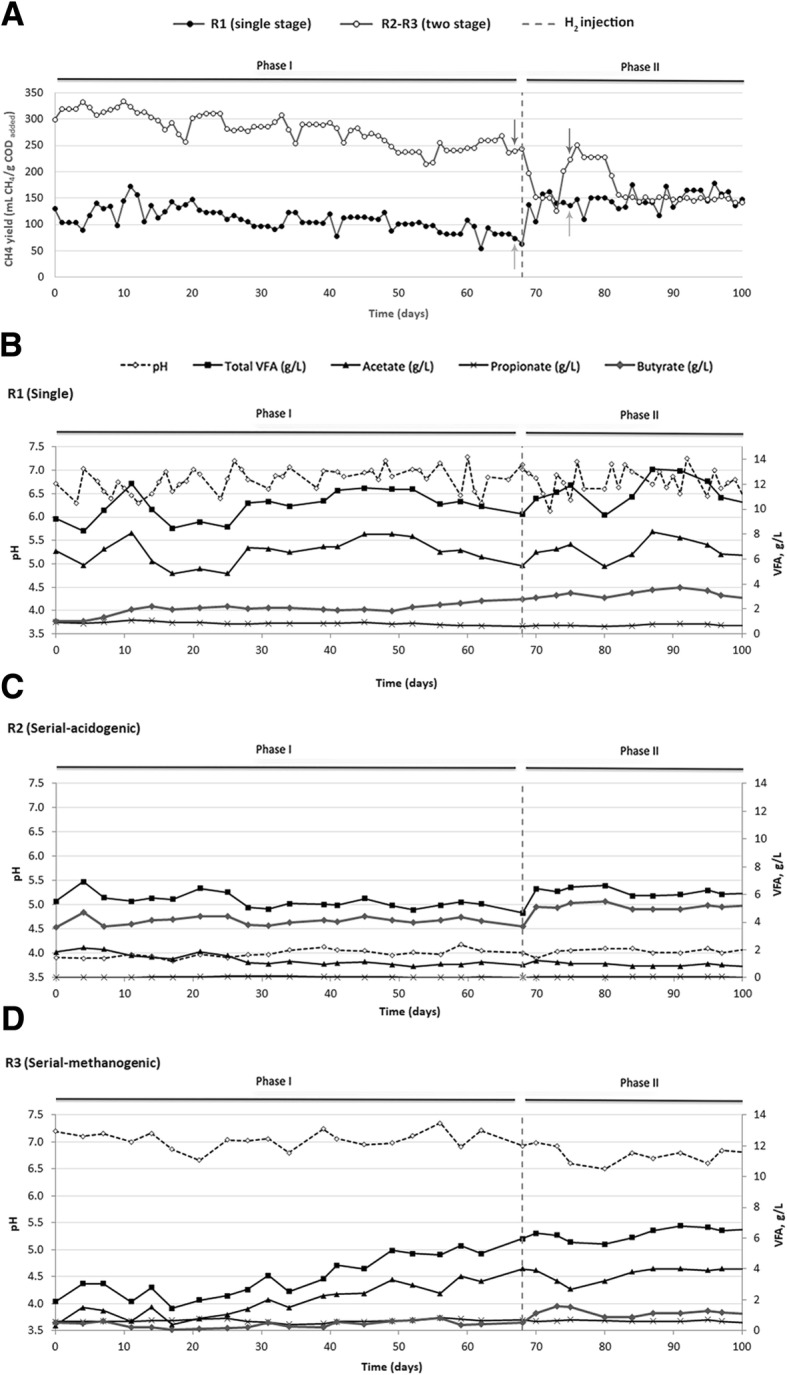
CH_4_ yield of the two configurations (**a**), pH and VFAs trends in R1 (**b**), R2 (**c**) and R3 (**d**), before (phase I) and after (phase II) H_2_ injection. The orange and green arrows highlight the DNA/RNA sampling points for the single and the two-stage configuration, respectively

Metagenomic and metatranscriptomic investigations were performed at two time points; the first point referred to the reactors’ steady-state performance before H_2_ injection (phase I) and the second occurred 1 week after H_2_ injection (phase II). To verify the stability of the microbial community during the reactors’ stable operation, an additional set of metagenomic samples was collected from R1 and R3 at multiple time points and was analyzed using 16S rRNA gene amplicon sequencing. The overview of the sequencing depth obtained with the different NGS data type showed that the microbial community under consideration was well captured (Additional file 1: Table S1). OTUs taxonomy showed that the biological process was adequately captured in terms of microbial composition; results from beta diversity demonstrated that the microbial community was stable during time, and thus, the selected point chosen for in depth analysis was representative of the steady-state period (Additional file 1: Table S2 and Figure S1). In particular, the overall OTUs’ taxonomic distribution in both R1 and R3 was in agreement with the profile of the reconstructed MAGs (Additional file 1: Table S2). PCoA results and OTUs relative abundances (i.e., 1.5 average fold change) revealed negligible variations among the different time points in R1 (Additional file 1: Table S2 and Figure S2). Regarding the reactor R3, the dominant OTUs abundances were coherent with MAGs coverages (Fig. 2a and Additional file 1: Table S2). The observed differences in the PCoA results were mainly attributed to the microbial diversity of a minor subset of OTUs in the middle sampling point. Considering the results from the biochemical parameters, the reactor operation was stable, indicating that this OTUs subset was not primarily involved in the methanation process.

The reconstructed MAGs identified in the microbial community represented more than 60% of the entire microbiome. Therefore, the results from the current work covered successfully the majority of the transcriptional changes occurring in the reactors excluding only a minor fraction of the information present in the shotgun reads. Moreover, the total number of protein-encoding genes identified in the assembly was slightly higher than 196,000, out of which 80% had at least 1 read in 1 of the samples examined and 27% had 10 or more reads. Consequently, the outcomes of the identified genes confirm that the transcriptional study was representing the expression of a considerable fraction of the total genes in the microbiome.

In order to acquire a global overview, analysis of the total expressed genes (not assigned to MAGs) was carried out considering COG classification (Additional file 1: Figure S3, Additional file 2: Dataset S3). In both reactor configurations, the most differentially expressed categories (excluding R and S categories, representing the general and unknown functions, respectively) belonged to the carbohydrate and amino acid transports and metabolisms. However, a high fraction of genes within the C category (energy production and conversion) was also differentially expressed in both single and two-stage reactors. Analysis of the expressed genes was subsequently performed in a genome-centric perspective to decipher the roles of the individual MAGs. The investigation was focused on the most abundant and active species, having more than 1000 expressed genes after H_2_ injection. However, the analysis was exceptionally expanded to two MAGs (*Methanothermobacter wolfeii* UC0008 and *Tepidanaerobacter acetatoxydans* UC0018) that were considered of particular interest, despite the fact that they showed less than 1000 expressed genes.

### Single-stage reactor: power-to-methane

The single-stage reactor (R1) exhibited a pH trend ranging between 6.3 and 7.3 during phase I (Fig. 2b). Total VFAs were highly concentrated (9.7 ± 1.1 g/L) and composed mainly of acetate (6.1 ± 1.0 g/L) (Fig. 2b). These conditions inhibited the activity of methanogenic archaea, resulting in a CH_4_ yield equal to 31% of the theoretical value, which is 350 mL CH_4_/g COD (Fig. 2a and Table 1). High VFAs concentrations lower the pH of the reactor, and thus, concomitantly lead to alteration of the microbial activities [37]. This effect is especially evident during the anaerobic digestion of acidic substrates characterized by poor buffering capacity [10]. In particular, methanogens are the most sensitive species to over acidification events, since their optimal growth rate ranges between the pH values of 6.5 and 8.5 [38]. Total VFAs increased by ~ 1 g/L in phase II, mainly due to higher butyrate concentration (Fig. 2b). This increase could be caused by the high acetate levels present in this reactor (6.7 ± 0.8 g/L), which may have hampered syntrophic butyrate oxidation [39]. Despite the further over acidification during phase II, the CH_4_ yield in R1 increased by 10% compared to the previous experimental phase (Fig. 2a and Table 1), indicating a positive effect of H_2_ injection on the methanogenic consortia.

**Table 1:** Reactors’ performance at phase I (steady state, before H_2_ injection) and phase II (1 week after H_2_ injection)

Reactor configuration	Phase I (pre-H_2_)			Phase II (post-H_2_)					
	CH_4_ yield (mL CH_4_/g COD_added_)	CH_4_ (%)	CO_2_ (%)	CH_4_ yield (mL CH_4_/g COD_added_)	CH_4_ (%)	CO_2_ (%)	H_2_ (%)	H_2_ consumption rate (mL/L day)	CO_2_ conversion rate (mL/L day)
Single stage	110 ± 21	44.6 ± 0.1	55.4 ± 0.1	142 ± 16	51.6 ± 0.1	23.0 ± 0.1	25.4 ± 0.1	648 ± 24	182 ± 56
Two-stage	276 ± 34	57.3 ± 0.1	42.7 ± 0.1	152 ± 16	39.7 ± 0.1	30.0 ± 0.1	30.3 ± 0.1	303 ± 64	173 ± 35

**Fig. 3:**
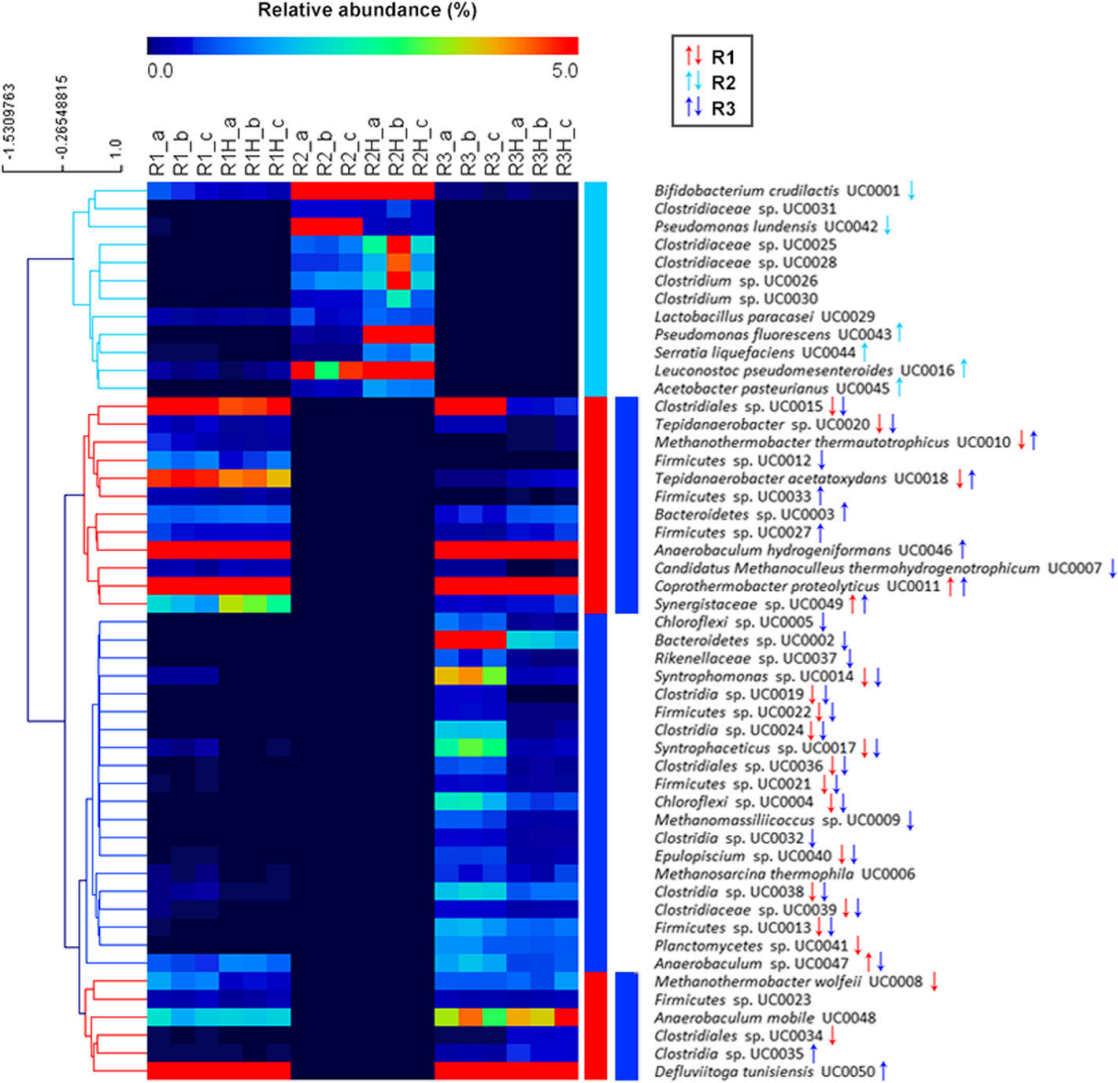
Heat map of relative abundance of the 50 MAGs (R1 and R1H: single stage pre- and post-H_2_, respectively; R2 and R2H: acidogenic reactor of the two-stage pre- and post-H_2_, respectively; R3 and R3H: methanogenic reactor of the two-stage pre- and post-H_2_, respectively; **a**–**c**: replicates). Up and down arrows indicate the statistically significant shifts in abundance of the MAGs (increase and decrease, respectively) between the two conditions (pre-/post-H_2_)

**Fig. 4:**
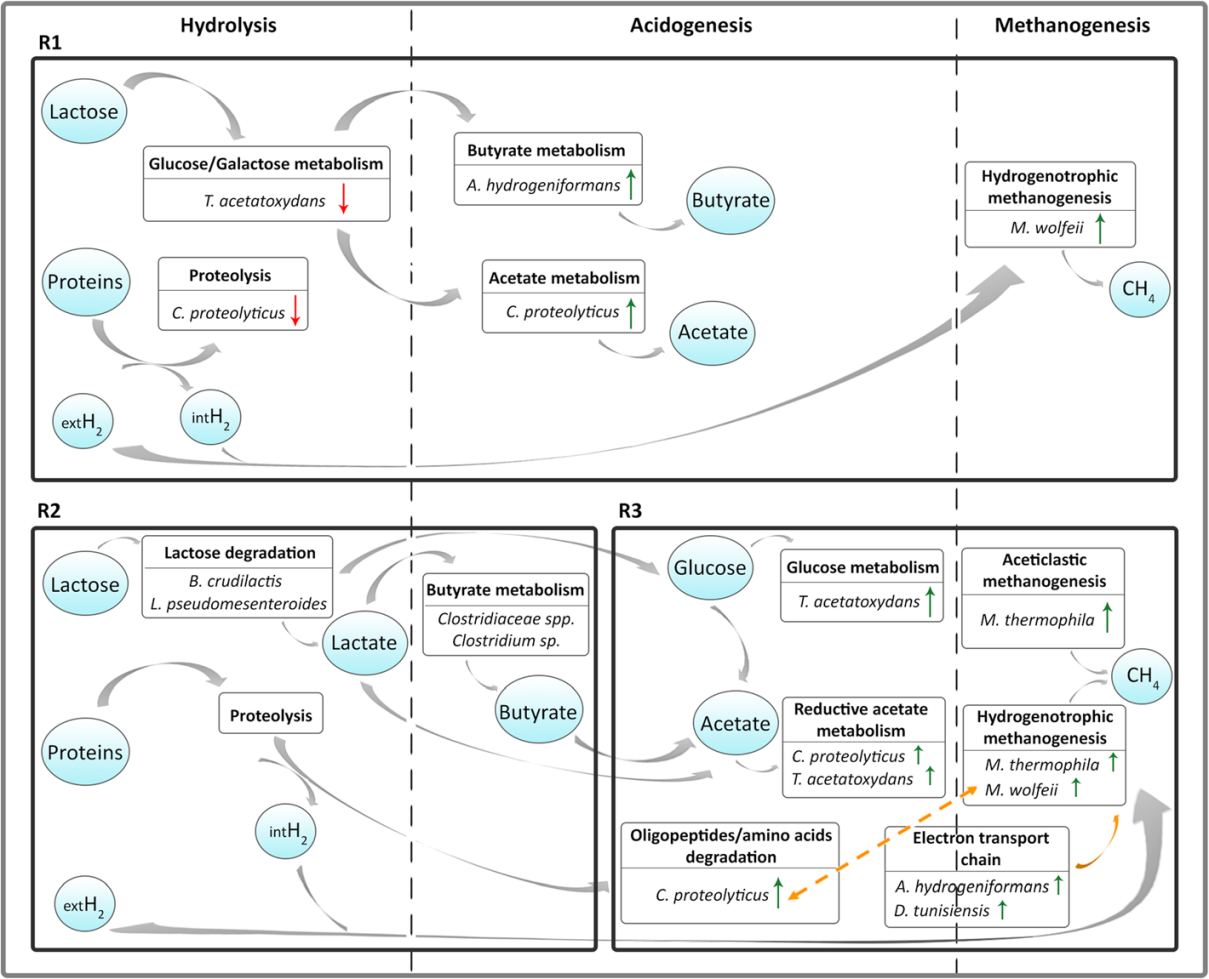
Schematic representation describing the main degradation pathways of the substrates and the responsible MAGs. Green and red arrows indicate pathways enriched with up- and downregulated genes, respectively. Orange arrow indicates the connection between MAGs which mostly upregulated electron transport chain mechanisms, and hydrogenotrophic archaea. Orange dashed arrow specifically highlights the proposed syntrophic association between *Coprothermobacter proteolyticus* UC0011 and *Methanothermobacter wolfeii* UC0008. Metabolic representation of R2 was based on the change in abundance of the indicated MAGs, while for R1 and R3 it was based on gene expression data. *R1* single-stage reactor, *R2* acidogenic reactor of the two-stage configuration, *R3* methanogenic reactor of the two-stage configuration, *extH*_2_ external hydrogen, *intH*_2_ internal hydrogen

**Fig. 5:**
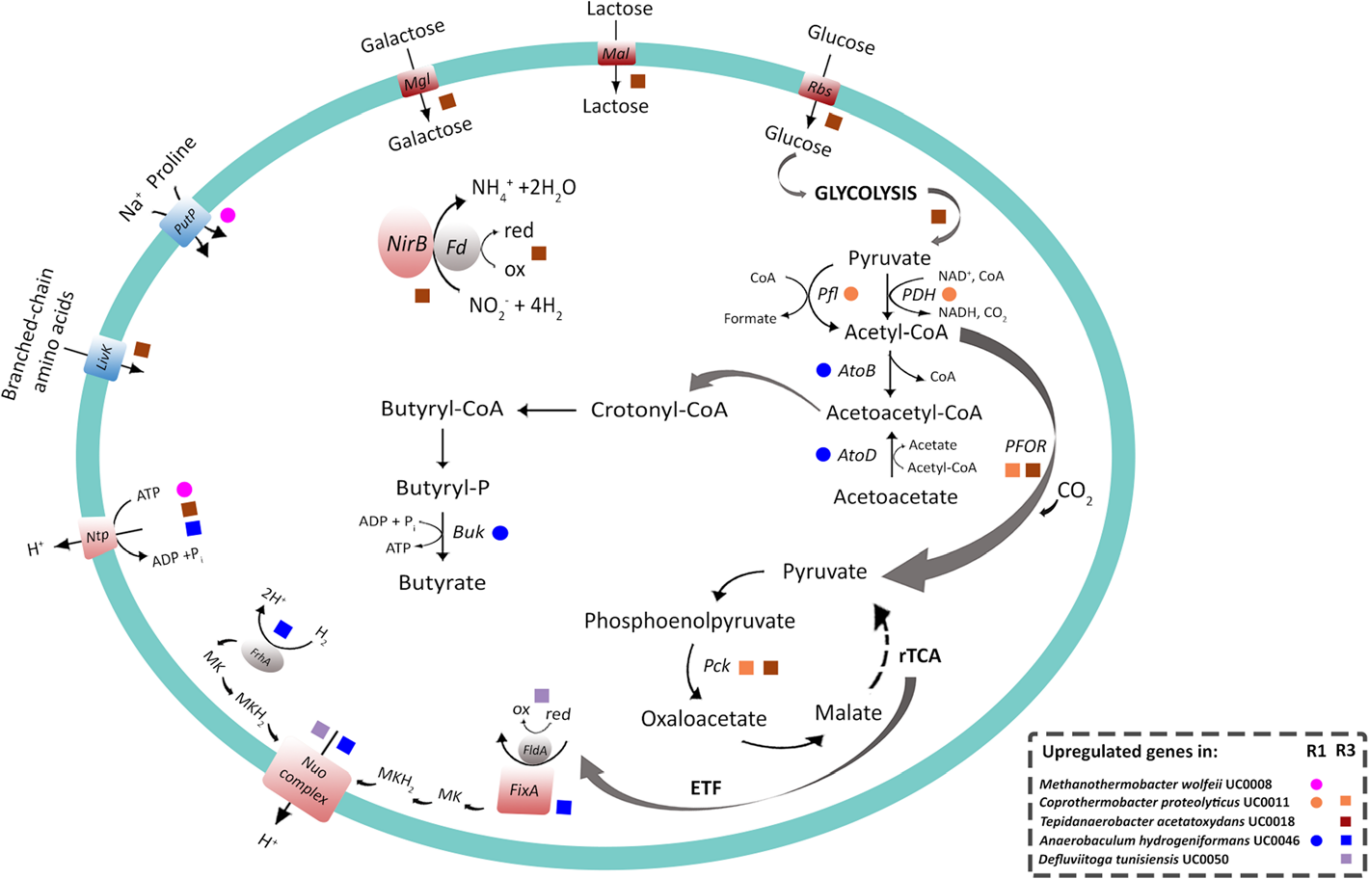
Main upregulated genes after H_2_ injection by the selected MAGs (indicated with colored dots and squares) in R1 (single-stage reactor) and R3 (methanogenic reactor of the two-stage configuration). *rTCA* reductive tricarboxylic acid cycle, *ETF* electron transfer flavoprotein

Three MAGs were identified as dominant (77% of the microbial community) in R1 during phase I, specifically *Coprothermobacter proteolyticus* UC0011, *Anaerobaculum hydrogeniformans* UC0046, and *Defluviitoga tunisiensis* UC0050 (Fig. 3, Additional file 1: Figure S4, Additional file 2: Dataset S1). This microbial core reached 85% of relative abundance after H_2_ injection, with *C*. *proteolyticus* UC0011 as the dominant species (61% relative abundance) (Fig. 3, Additional file 1: Figure S4, Additional file 2: Dataset S1). These results highlight the strong microbial selection operated by both the feed characteristics such as the acidic pH and low buffer capacity, and the increased H_2_ partial pressure inside the reactor. A significative correlation between *C*. *proteolyticus* UC0011 in phase II and H_2_ content in the reactor was also highlighted by statistical analysis (Additional file 1: Figure S5). Transcriptional data showed that *C*. *proteolyticus* UC0011 responded to H_2_ addition by differentially expressing genes related to carbon metabolism, specifically the pyruvate metabolic pathway (Table 2 and Fig. 4). Genes associated with the pyruvate dehydrogenase complex and pyruvate-formate lyase (Aco, Ace, and Pfl), both involved in acetyl-CoA production, increased their expression by ~ 3-fold in *C*. *proteolyticus* UC0011 (Fig. 5 and Additional file 2: Dataset S2). This upregulation suggests that *C*. *proteolyticus* UC0011 is involved in the acetate accumulation observed in R1 (Fig. 4 and Fig. 5). In contrast, expression of the ATP-dependent protease Clp was inhibited by ~ 4-fold in *C*. *proteolyticus* UC0011 (Fig. 4 and Additional file 2: Dataset S2), indicating a specific repression of the proteolytic activity of this enzyme, which causes H_2_ release [40, 41].

**Table 2:** Number of differentially expressed (DE) genes per KEGG category of selected MAGs in R1 (single-stage configuration) and R3 (methanogenic reactor of the two-stage configuration)

MAG	*M*. *thermophila* UC0006	*M*. *wolfeii* UC0008		*C*. *proteolyticus* UC0011		*T*. *acetatoxydans* UC0018		*A*. *hydrogeniformans* UC0046	*D*. *tunisiensis* UC0050	
	Reactor									
KEGG category	R3	R1	R3	R1	R3	R1	R3	R1	R1	R3
ABC transporters	3	5	4	2	0	7	10	4	0	11
Amino acids metabolism	19	5	0	0	5	5	35	8	0	21
Bacterial chemotaxis	0	0	0	0	0	3	8	1	0	5
Biosynthesis of amino acids	43	4	3	0	4	3	37	2	0	39
Biosynthesis of antibiotics	18	8	4	3	10	4	28	1	0	6
Biosynthesis of secondary metabolites	13	8	3	3	5	4	28	0	0	20
Butanoate metabolism	3	1	1	1	6	0	3	0	0	7
Carbon fixation pathways in prokaryotes	3	4	2	0	7	0	5	1	0	5
Carbon metabolism	18	21	12	4	11	4	19	1	0	11
Citrate cycle (TCA cycle)	1	4	1	2	9	0	6	0	0	5
Fatty acid metabolism	0	0	0	0	0	0	0	2	0	1
Flagellar assembly	0	0	0	0	0	0	7	0	0	0
Galactose metabolism	1	0	0	2	0	5	6	0	0	1
Glycolysis/Gluconeogenesis	1	1	1	2	7	4	11	0	0	5
Metal transport system	6	0	5	0	1	0	6	0	0	8
Methane metabolism	21	64	14	0	0	0	7	0	0	3
Nitrogen metabolism	5	0	0	0	1	1	3	1	0	1
Peptidoglycan biosynthesis	0	0	0	0	1	0	3	0	0	2
Propanoate metabolism	1	0	1	1	1	1	2	2	0	1
Purine metabolism	0	4	0	1	0	1	0	0	0	0
Pyrimidine metabolism	6	5	1	1	3	0	0	0	0	1
Pyruvate metabolism	0	3	0	3	0	1	0	0	0	0
Quorum sensing	4	0	0	1	2	0	13	0	3	7
Reductive acetyl-CoA pathway (Wood-Ljungdahl)	1	1	0	0	0	2	0	0	0	0
Ribosome	28	25	5	3	1	0	16	0	0	5
Sugar, amino acid and oligo-peptide transport system	0	0	0	0	1	0	25	0	0	1
Triacylglycerol biosynthesis	1	0	0	0	0	0	1	0	0	2
Two-component system	2	0	0	0	0	0	9	0	0	5

Analysis of *A*. *hydrogeniformans* UC0046 revealed the differential expression of genes encoding ABC transporters related to amino acid translocation across the plasma membrane (Table 2 and Fig. 4). Expression of acetyl-CoA acetyltransferase (AtoB), acetyl-CoA:acetoacetyl-CoA transferase (AtoD), and butyrate kinase (Buk) increased ~ 3-fold, all involved in butyrate metabolism (Fig. 5 and Additional file 2: Dataset S2). Indeed, Ato enzymes participate in the degradation of acetoacetate intermediate, which can be subsequently funneled to the central energy-gaining step, where crotonyl-CoA is converted to butyryl-CoA [42]. Moreover, the gene coding for Buk enzyme, which catalyzes the final step for butyrate formation, is frequently used as biomarker for the identification of butyrate-producing communities [43]. Therefore, these results indicate that *A*. *hydrogeniformans* UC0046 contributes to the increased butyrate concentration found in R1 after H_2_ injection.

Only 11 genes of *D*. *tunisiensis* UC0050 were differentially expressed after H_2_ addition, and 3 were involved in quorum sensing activities (Table 2). RpoD (sigma 70) (Additional file 2: Dataset S2) is the main bacterial sigma factor responsible for housekeeping gene transcription [44], and showed decreased expression. This regulation pattern suggests an inhibition of basal gene expression in this species during phase II.

The known syntrophic acetate-oxidizing bacterium (SAOB) *T*. *acetatoxydans* UC0018 [41] slightly decreased in abundance after H_2_ addition (Fig. 3, Additional file 1: Figure S4, Additional file 2: Dataset S1). Transcriptomic data showed that *T*. *acetatoxydans* UC0018 differentially expressed genes encoding ABC transporters and enzymes involved in amino acid and sugar metabolism (Table 2 and Fig. 4). Sugar intake was decreased by the downregulation of specific ABC transporters (Mgl permease) as well as genes related to glucose and galactose metabolism (*nag* sugar kinase, *fruK*, *fba*) by 4- and 8-fold, respectively (Additional file 2: Dataset S2). This regulation suggests the existence of a “feedback mechanism” to limit excessive acetate production via sugar catabolism.

Regarding the methanogenic consortia, there was a clear dominance of one archaeal species, the hydrogenotrophic *M*. *wolfeii* UC0008, which was reduced in abundance by half after H_2_ injection (Fig. 3, Additional file 1: Figure S4, Additional file 2: Dataset S1). A significative reduction was also observed for the less abundant hydrogenotrophic *Methanothermobacter thermautotrophicus* UC0010 (Fig. 3, Additional file 1: Figure S4, Additional file 2: Dataset S1). The growth inhibition of Archaea may be partly due to the low alkalinity intrinsic to cheese whey permeate, along with the increased acidification of the system following H_2_ addition (Fig. 2b) [38]. Despite this inhibition, H_2_ injection induced a significant upregulation of the hydrogenotrophic methanogenesis pathway in *M*. *wolfeii* UC0008 (Additional file 1: Figure S6). Such transcriptional behavior led to increased CH_4_ content in the biogas, with a ~ 54% CO_2_ conversion efficiency (Table 1).

The high accumulation of acetate measured in this reactor could also be related to the lack of aceticlastic methanogens, whose growth was probably not favored by the conditions established in the single-stage configuration. In addition to the difficulty in maintaining the pH in a proper range for methanogenic growth, toxicity related to the accumulation of cations (i.e., potassium) or lipids has been previously hypothesized [10].

### Two-stage configuration, acidogenic reactor: power-to-chemicals

The hypothesis for applying the H_2_ only into the acidogenic reactor (R2) of the two-stage configuration was that it could better withstand a potential pH increment that could be caused by the transformation of CO_2_ into methane. This reactor indeed maintained a stable pH (~ 4) throughout the process. The main VFA produced in R2 was butyrate (3.9 ± 0.7 g/L), which increased by ~ 1 g/L after H_2_ injection (Fig. 2c). *Bifidobacterium crudilactis* UC0001 was the dominant species inhabiting this reactor, and it showed a change in abundance after H_2_ addition, decreasing from 82 to 52% of the total microbiome (Fig. 3, Additional file 1: Figure S4, Additional file 2: Dataset S1). In contrast, the heterofermentative lactic acid bacteria *Leuconostoc pseudomesenteroides* UC0016 strongly increased in abundance (~ 3-fold). This variation could be related to the higher butyrate concentration present in R2 during phase II (Fig. 2c), since the lactose fermentation to lactate by *L*. *pseudomesenteroides* UC0016 can enhance the cross-feeding of *Clostridiales* species involved in the conversion of lactic acid to butyrate [45]. It was indeed observed a ~ 4-fold increase of *Clostridiaceae* sp. UC0025, *Clostridiaceae* sp. UC0028, and *Clostridium* sp. UC0030 during phase II (Fig. 3, Additional file 1: Figure S4, Additional file 2: Dataset S1). Moreover, butyrate production by clostridial-type fermentation is also known to be favored under high H_2_ partial pressures [46–50], as can occur during exogenous H_2_ injection in R2. The significative effect of butyrate increase in phase II on microbial distribution was also evidenced by statistical analysis (Additional file 1: Figure S5).

### Two-stage configuration, methanogenic reactor: power-to-chemicals

The methanogenic reactor of the two-stage configuration (R3) maintained the pH between 6.7 and 7.5 during phase I, exhibiting lower accumulation of total VFAs (primarily acetate) than the single-stage configuration (3.4 ± 1.3 g/L) (Fig. 2d). These operating conditions resulted in a CH_4_ yield equal to 80% of the theoretical value (Fig. 2a, d). However, H_2_ addition induced an increase in total VFAs (primarily butyrate), which doubled in concentration to 6.1 ± 0.3 g/L (Fig. 2d). The CH_4_ yield was highly reduced under these conditions (Fig. 2a and Table 1), and the increased butyrate and acetate levels along with the decreased CH_4_ content seen in phase II indicate that CO_2_ fixation toward SCFAs overtook the methanation pathways. The most abundant MAGs were the same as those found in the single stage R1, specifically *C*. *proteolyticus* UC0011, *A*. *hydrogeniformans* UC0046, and *D*. *tunisiensis* UC0050, which accounted for 47% of the microbiome in the reactor (Fig. 3, Additional file 1: Figure S4, Additional file 2: Dataset S1). This microbial core reached 81% of relative abundance after H_2_ injection, and *D*. *tunisiensis* UC0050 was the dominant species (54% of the total community) (Fig. 3, Additional file 1: Figure S4, Additional file 2: Dataset S1). Transcriptomic data indicated that *D*. *tunisiensis* UC0050 differentially expressed genes involved in carbon metabolism and fixation pathways for energy production (Table 2 and Fig. 4). It was observed a ~ 4-fold increase in NADH:ubiquinone oxidoreductase expression (the NuoE subunit, forming the NADH dehydrogenase module), which may function as electron acceptor for the also consistently highly expressed flavodoxin FldA (Fig. 5 and Additional file 2: Dataset S2). The NADH:ubiquinone oxidoreductase enzyme is indeed a proton pump (also known as complex I), which couples electron transfer with the translocation of four protons through the membrane [51]. This electron flow can be mediated via a reduced flavodoxin, such as FldA, which acts as intermediate between central carbon metabolism (e.g., TCA cycle) and complex I [52]. Thus, the upregulation of these genes suggests an increased activity of the electron transfer chain via H_2_ oxidation [51], and may be involved in syntrophic relationships with hydrogenotrophic species throughout the increased proton extrusion from the cell.

Similarly to *D*. *tunisiensis* UC0050, *C*. *proteolyticus* UC0011 also differentially expressed genes related to carbon fixation pathways (Table 2 and Fig. 4). Specifically, *C*. *proteolyticus* UC0011 boosted the reductive tricarboxylic acid cycle (rTCA) by a ~ 6-fold increase in the expression of pyruvate:ferredoxin oxidoreductase (PFOR) and phosphoenolpyruvate carboxykinase (Pck) (Fig. 5 and Additional file 2: Dataset S2). Such regulation indicates an uptake of the excess acetate for pyruvate production, from which other central metabolic intermediates can be formed. *C*. *proteolyticus* UC0011 also regulated genes involved in amino acids metabolism, including a ~ 4-fold upregulation of the ATP-dependent protease Clp, along with enzymes metabolizing various amino acids (arginine, alanine, glutamate, tryptophan, aspartate) (Fig. 4, Additional file 2: Dataset S2). Since H_2_ is one of the main products derived from proteins and amino acids degradation by *C*. *proteolyticus* [40, 41], it cannot be excluded that this microbial species can form syntrophic association with hydrogen-scavenger microorganisms, such as the hydrogenotrophic methanogen *M*. *wolfeii* UC0008. Previous studies pointed out a synergistic effect operated by the co-existence of proteolytic anaerobes and hydrogen-consuming methanogens, revealing an augmented cell growth and protein degradation efficiency [53]. A partnership between *C*. *proteolyticus* and archaeal species belonging to the *Methanothermobacter* genus has also been recently proposed [54, 55].

*A*. *hydrogeniformans* UC0046 responded to H_2_ injection by upregulating a H^+^-ATPase (NtpB) (Fig. 5 and Additional file 2: Dataset S2) that extrudes protons through ATP hydrolysis, and by downregulating the expression of the Na^+^/proline symporter (PutP) and Na^+^/H^+^ antiporters (MnhC, NhaC) (Additional file 2: Dataset S2). These mechanisms are used by various anaerobic bacteria to regulate internal pH and to control the transmembrane electrochemical gradient [56]; however, a syntrophic mechanism also cannot be excluded for this species. Additionally, *A*. *hydrogeniformans* UC0046 upregulated the coenzyme F420-reducing hydrogenase (FrhA), the electron transfer flavoprotein (ETF: FixA), and the NADH:ubiquinone oxidoreductase (NuoE), all known to be involved in mechanisms of electron flow and energy production [51, 52] (Fig. 5 and Additional file 2: Dataset S2). As for *D*. *tunisiensis* UC0050, the upregulation of these genes suggests an involvement of *A*. *hydrogeniformans* UC0046 in syntrophic relationships with hydrogen-scavenger microorganisms.

The less abundant SAOB *T*. *acetatoxydans* UC0018 increased by almost 8-fold after H_2_ injection (Fig. 3, Additional file 1: Figure S4, Additional file 2: Dataset S1). There was an upregulation of glucose metabolism (FruK, Fba), as well as sugar and branched-chain amino acid ABC transporters (Rbs, Mgl, and LivK) in this species (Fig.4, Fig. 5, Additional file 2: Dataset S2). *T*. *acetatoxydans* UC0018 also upregulated the rTCA key enzymes pyruvate:ferredoxin oxidoreductase and phosphoenolpyruvate carboxykinase (PorA and PckA) by 8- and 4-fold, respectively (Fig. 5 and Additional file 2: Dataset S2), indicating an acetate uptake probably aimed to increase the energy store capacity. It is indeed known that the utilization of the TCA cycle in the reductive direction by many autotrophic anaerobes is aimed at producing metabolic intermediates via acetyl-CoA incorporation [57]. The rTCA upregulation seen in *T*. *acetatoxydans* UC0018 indicates the different metabolic direction taken by *T*. *acetatoxydans*, which did not act as a SAO by upregulating enzymes for acetate oxidation. A significative correlation between *T*. *acetatoxydans* UC0018 in phase II and acetate concentration in the reactor was also indicated by statistical analysis (Additional file 1: Figure S5).

The archaeal consortium was composed of three equally abundant methanogens: *M*. *wolfeii* UC0008, the generalist *Methanosarcina thermophila* UC0006 [58], and the methylotrophic *Methanomassiliicoccus* sp. UC0009 [59]. Only the latter decreased in abundance after H_2_ injection (Fig. 3, Additional file 1: Figure S4, Additional file 2: Dataset S1), showing a ~ 4-fold reduction and indicating that this species may be more sensitive to the new condition. The remarkable decrease of *Methanomassiliicoccus* sp. UC0009 and therefore the CH_4_ produced by the methylotrophic pathway could be also one determinant of the lower methanation seen in R3 after H_2_ addition. Additionally, although *M*. *thermophila* UC0006 and *M*. *wolfeii* UC0008 remained quantitatively stable and upregulated the aceticlastic (*M*. *thermophila* UC0006) and hydrogenotrophic pathways in phase II (Additional file 1: Figures S7 and S8), the drop in CH_4_ content seen in R3 was unchanged (Fig. 2a and Table 1). However, it is worth to highlight that the presence of *M*. *thermophila* UC0006 in R3 may have allowed the lower accumulation of acetate compared to the single-stage configuration.

### Simulation of hydrogen utilization routes in R1 and R3

The same bacterial species had different regulatory responses in the two reactors. This diverse regulation could be due to the reactor configurations, resulting in different physicochemical conditions, and consequently different H2 utilization capability.

To support the experimental findings based on gene expression results (Fig. 4 and Fig. 5), which showed the main metabolic pathways undertaken by the MAGs, a computational model of the two reactor configurations was also developed. Moreover, mass balance calculations contributed to clarify the processes occurring in the reactors after H_2_ injection (Additional file 1). It was found that approximately 40% of the H_2_ moles injected in the single-stage configuration (R1) per day were effectively utilized to produce CH_4_. Software simulation results for R1 showed trends similar to those obtained experimentally. In particular, both simulated methane production and total VFA concentration curves agreed with the measured values, although being slightly lower (Additional file 1: Figures S9 and S10). However, the remaining 60% of the added H_2_ was not enough to account for the butyrate increase seen in the same reactor (Additional file 1). Therefore, the additional utilization of internal H_2_ produced by lactose fermentation to acetate and butyrate should be considered. The most reasonable hypothesis in terms of demand for indigenous H_2_ moles suggests that propionate reduction increased butyrate (Additional file 1). Concerning the acidogenic reactor of the two-stage configuration (R2), the fractions of exogenous H_2_ moles utilized for the butyrate augmentation were ~ 97%, 60%, and 30%, based on the substrate reduced (CO_2_, acetate, and propionate, respectively). Since the amount of propionate in R2 was negligible and butyrate increase cannot be primarily based on CO_2_ reduction (considering the 30% residual hydrogen in the effluent gas from R3), the most probable explanation for butyrate production is via the acetate reduction. This was further confirmed by the slight decrease of acetate concentration in R2 (Fig. 2c). Additionally, the acetate rise seen in the methanogenic reactor of the same configuration (R3) during phase II mostly relied on butyrate oxidation (~ 60%), also considering the augmented butyrate feeding from R2. Overall, model simulations of the methane production and changes in total VFA concentration were in agreement with the above assumptions (Additional file 1: Figures S15 and S16). Finally, since only ~ 10% of the acetate rise in R3 could be explained via the acetogenic pathway, it is reasonable that the remaining 30% of the acetate mole increase was likely due to an accumulation effect, which may have inhibited the acetogenic pathway.

Computer-aided simulations, combined with mass balance calculations, indicate the most probable H_2_ availabilities in the two reactor configurations and therefore the different metabolic routes for H_2_ utilization used by the anaerobic digestion microbiome.

## Conclusions

H_2_ injection induced different transcriptional regulation responses in the same MAGs inhabiting the two reactors. Specifically, they favored methanation in the single-stage reactor (power-to-methane), and SCFAs production in the two-stage configuration (power-to-chemicals). The above finding was also confirmed by model simulations. Gene expression results revealed that *C*. *proteolyticus* UC0011 and *A*. *hydrogeniformans* UC0046 mainly upregulated pathways involved in acetate and butyrate production. However, a 7% increase in CH_4_ content in the biogas of the single-stage reactor was observed, mainly due to the dominant hydrogenotrophic *M*. *wolfeii* UC0008. In contrast, a doubling of total SCFAs by CO_2_ fixation was evidenced in the two-stage configuration, with *A*. *hydrogeniformans* UC0046 and *D*. *tunisiensis* UC0050 upregulating genes involved in electron transport chains. Interestingly, the SAOB *T*. *acetatoxydans* UC0018 did not act as acetate-oxidizer in either reactor configuration, but primarily inhibited sugar metabolism in the single stage and boosted acetate uptake via the reductive TCA cycle in the two-stage configuration. A putative syntrophism between *C*. *proteolyticus* UC0011 and *M*. *wolfeii* UC0008 was proposed in the serial reactor configuration.

## Additional files

Additional file 1: 16S rRNA gene amplicon results (**Table S2** and **Figures** **S1**-**S2**), differentially expressed genes not assigned to MAGs (**Figure S3**), relative abundance of the 50 MAGs (Figure S4), statistical analysis (Figure S5), methanogenic pathways regulated by the discussed archaeal MAGs (**Figures** **S5**-**S7**), simulation results (Figures S9-S19) and mass balance calculations. (DOCX 5847 kb)

Additional file 2: MAGs coverage (Dataset S1), differentially expressed genes assigned to MAGs (Dataset S2), total differentially expressed genes not assigned to MAGs (Dataset S3), KEGG pathways including a significant number of differentially expressed genes (Dataset S4). (XLSX 328 kb)

## Abbreviations

AD: Anaerobic digestion
COG: Clusters of orthologous groups
CSTR: Continuous stirred tank reactor
DE: Differentially expressed
HRT: Hydraulic retention time
KEGG: Kyoto Encyclopedia of Genes and Genomes
KO: KEGG orthology
MAG: Metagenome assembled genome
OLR: Organic loading rate
rTCA: Reductive tricarboxylic acid cycle
SAOB: Syntrophic acetate-oxidizing bacteria
SCFA: Short-chain fatty acid
VFA: Volatile fatty acid

## References

[1] World Wind Energy Association (WWEA): WWEC2017: Key Statistics of World Wind Energy Report published. http://www.wwindea.org/2017-statistics/ (accessed on June 2018).

[2] Turner J, Sverdrup G, Mann MK, Maness P-C, Kroposki B, Ghirardi M (2008). Renewable hydrogen production. Int J Energy Res.

[3] Angelidaki I, Treu L, Tsapekos P, Luo G, Campanaro S, Wenzel H (2018). Biogas upgrading and utilization: current status and perspectives. Biotechnol Adv.

[4] Wang K, Yin J, Shen D, Li N (2014). Anaerobic digestion of food waste for volatile fatty acids (VFAs) production with different types of inoculum: effect of pH. Bioresour Technol.

[5] Treu L, Kougias PG, de Diego-Díaz B, Campanaro S, Bassani I, Fernández-Rodríguez J (2018). Two-year microbial adaptation during hydrogen-mediated biogas upgrading process in a serial reactor configuration. Bioresour Technol.

[6] Treu L, Campanaro S, Kougias PG, Sartori C, Bassani I, Angelidaki I (2018). Hydrogen-fueled microbial pathways in biogas upgrading systems revealed by genome-centric metagenomics. Front Microbiol.

[7] Lee WS, Chua ASM, Yeoh HK, Ngoh GC (2014). A review of the production and applications of waste-derived volatile fatty acids. Chem Eng J.

[8] Li X, Chen H, Hu L, Yu L, Chen Y, Gu G (2011). Pilot-scale waste activated sludge alkaline fermentation, fermentation liquid separation, and application of fermentation liquid to improve biological nutrient removal. Environ Sci Technol.

[9] Chen H, Meng H, Nie Z, Zhang M (2013). Polyhydroxyalkanoate production from fermented volatile fatty acids: effect of pH and feeding regimes. Bioresour Technol.

[10] Fontana A, Campanaro S, Treu L, Kougias PG, Cappa F, Morelli L (2018). Performance and genome-centric metagenomics of thermophilic single and two-stage anaerobic digesters treating cheese wastes. Water Res.

[11] Imelfort M, Parks D, Woodcroft BJ, Dennis P, Hugenholtz P, Tyson GW (2014). GroopM: an automated tool for the recovery of population genomes from related metagenomes. PeerJ.

[12] Kang DD, Froula J, Egan R, Wang Z (2015). MetaBAT, an efficient tool for accurately reconstructing single genomes from complex microbial communities. PeerJ.

[13] Campanaro S, Treu L, Kougias PG, De Francisci D, Valle G, Angelidaki I (2016). Metagenomic analysis and functional characterization of the biogas microbiome using high throughput shotgun sequencing and a novel binning strategy. Biotechnol Biofuels.

[14] Bassani I, Kougias PG, Treu L, Angelidaki I (2015). Biogas upgrading via hydrogenotrophic methanogenesis in two-stage continuous stirred tank reactors at mesophilic and thermophilic conditions. Environ Sci Technol.

[15] Bassani I, Kougias PG, Angelidaki I (2016). In-situ biogas upgrading in thermophilic granular UASB reactor: key factors affecting the hydrogen mass transfer rate. Bioresour Technol.

[16] Treu L, Campanaro S, Kougias PG, Zhu X, Angelidaki I (2016). Untangling the effect of fatty acid addition at species level revealed different transcriptional responses of the biogas microbial community members. Environ Sci Technol..

[17] Bolger AM, Lohse M, Usadel B (2014). Trimmomatic: a flexible trimmer for Illumina sequence data. Bioinformatics.

[18] Takahashi S, Tomita J, Nishioka K, Hisada T, Nishijima M (2014). Development of a prokaryotic universal primer for simultaneous analysis of bacteria and archaea using next-generation sequencing. PLoS One.

[19] Hyatt D, Locascio PF, Hauser LJ, Uberbacher EC (2012). Gene and translation initiation site prediction in metagenomic sequences. Bioinformatics.

[20] Galperin MY, Makarova KS, Wolf YI, Koonin EV (2015). Expanded microbial genome coverage and improved protein family annotation in the COG database. Nucleic Acids Res.

[21] Finn RD, Bateman A, Clements J, Coggill P, Eberhardt RY, Eddy SR (2014). Pfam: the protein families database. Nucleic Acids Res.

[22] Kanehisa M, Sato Y, Morishima K (2016). BlastKOALA and GhostKOALA: KEGG tools for functional characterization of genome and metagenome sequences. J Mol Biol.

[23] Huerta-Cepas J, Szklarczyk D, Forslund K, Cook H, Heller D, Walter MC (2016). EGGNOG 4.5: a hierarchical orthology framework with improved functional annotations for eukaryotic, prokaryotic and viral sequences. Nucleic Acids Res.

[24] Langmead B, Salzberg S (2012). Fast gapped-read alignment with bowtie 2. Nat Methods.

[25] Anders S, Pyl PT, Huber W (2015). HTSeq-A Python framework to work with high-throughput sequencing data. Bioinformatics.

[26] Campanaro S, Treu L, Kougias PG, Luo G, Angelidaki I (2018). Metagenomic binning reveals the functional roles of core abundant microorganisms in twelve full-scale biogas plants. Water Res.

[27] Saeed AI, Sharov V, White J, Li J, Liang W, Bhagabati N (2003). TM4: a free, open-source system for microarray data management and analysis. BioTechniques.

[28] Zhou X, Lindsay H, Robinson MD (2014). Robustly detecting differential expression in RNA sequencing data using observation weights. Nucleic Acids Res.

[29] Kanehisa M, Goto S, Sato Y, Furumichi M, Tanabe M (2012). KEGG for integration and interpretation of large-scale molecular data sets. Nucleic Acids Res.

[30] Treu L, Campanaro S, Nadai C, Toniolo C, Nardi T, Giacomini A (2014). Oxidative stress response and nitrogen utilization are strongly variable in Saccharomyces cerevisiae wine strains with different fermentation performances. Appl Microbiol Biotechnol.

[31] Dixon P (2003). VEGAN, a package of R functions for community ecology. J Veg Sci.

[32] Torondel B, Ensink JHJ, Gundogdu O, Ijaz UZ, Parkhill J, Abdelahi F (2016). Assessment of the influence of intrinsic environmental and geographical factors on the bacterial ecology of pit latrines. Microb Biotechnol.

[33] Angelidaki I, Ellegaard L, Ahring BK (1999). A comprehensive model of anaerobic bioconversion of complex substrates to biogas. Biotechnol Bioeng.

[34] Angelidaki I, Ellegaard L, Ahring BK (1993). A mathematical model for dynamic simulation of anaerobic digestion of complex substrates: focusing on ammonia inhibition. Biotechnol Bioeng.

[35] Lovato G, Alvarado-Morales M, Kovalovszki A, Peprah M, Kougias PG, Rodrigues JAD (2017). In-situ biogas upgrading process: modeling and simulations aspects. Bioresour Technol.

[36] Kovalovszki A, Alvarado-Morales M, Fotidis IA, Angelidaki I (2017). A systematic methodology to extend the applicability of a bioconversion model for the simulation of various co-digestion scenarios. Bioresour Technol.

[37] Franke-Whittle IH, Walter A, Ebner C, Insam H (2014). Investigation into the effect of high concentrations of volatile fatty acids in anaerobic digestion on methanogenic communities. Waste Manag.

[38] Weiland P (2010). Biogas production: current state and perspectives. Appl Microbiol Biotechnol.

[39] Schink B, Stams AJM. The Prokaryotes: Prokaryotic Communities and Ecophysiology. In: Rosenberg E, DeLong EF, Lory S, Stackebrandt E, Thompson F, editors. Springer Berlin Heidelberg; 2013. p. 471–493.

[40] OLLIVIER B. M., MAH R. A., FERGUSON T. J., BOONE D. R., GARCIA J. L., ROBINSON R. (1985). Emendation of the Genus Thermobacteroides: Thermobacteroides proteolyticus sp. nov., a Proteolytic Acetogen from a Methanogenic Enrichment. International Journal of Systematic Bacteriology.

[41] Kersters I, Maestrojuan GM, Torck U, Vancanneyt M, Kersters K, Verstraete W (1994). Isolation of *Coprothermobacter proteolyticus* from an anaerobic digest and further characterization of the species. Syst Appl Microbiol.

[42] Vital M, Howe AC, Tiedje JM (2014). Revealing the bacterial butyrate synthesis pathways by analyzing (meta)genomic data. MBio.

[43] Vital M, Penton CR, Wang Q, Young VB, Antonopoulos DA, Sogin ML (2013). A gene-targeted approach to investigate the intestinal butyrate-producing bacterial community. Microbiome.

[44] Wösten MMSM (1998). Eubacterial sigma-factors. FEMS Microbiol Rev.

[45] Duncan SH, Louis P, Flint HJ (2004). Lactate-utilizing bacteria, isolated from human feces, that produce butyrate as a major fermentation product. Appl Environ Microbiol.

[46] Angenent LT, Karim K, Al-Dahhan MH, Wrenn BA, Domíguez-Espinosa R (2004). Production of bioenergy and biochemicals from industrial and agricultural wastewater. Trends Biotechnol.

[47] Girbal L, Croux C, Vasconcelos I, Soucaille P (1995). Regulation of metabolic shifts in *Clostridium acetobutylicum* ATCC 824. FEMS Microbiol Rev.

[48] Hallenbeck PC (2005). Fundamentals of the fermentative production of hydrogen. Water Sci Technol.

[49] Kraemer JT, Bagley DM (2007). Improving the yield from fermentative hydrogen production. Biotechnol Lett.

[50] Lee DJ, Show KY, Su A (2011). Dark fermentation on biohydrogen production: pure culture. Bioresour Technol.

[51] Weerakoon DR, Olson JW (2008). The *Campylobacter jejuni* NADH:ubiquinone oxidoreductase (complex I) utilizes flavodoxin rather than NADH. J Bacteriol.

[52] Sieber JR, Sims DR, Han C, Kim E, Lykidis A, Lapidus AL (2010). The genome of *Syntrophomonas wolfei*: new insights into syntrophic metabolism and biohydrogen production. Environ Microbiol.

[53] Stams AJM (1994). Metabolic interactions between anaerobic bacteria in methanogenic environments. Antonie Van Leeuwenhoek.

[54] Sasaki K, Morita M, Sasaki D, Nagaoka J, Matsumoto N, Ohmura N (2011). Syntrophic degradation of proteinaceous materials by the thermophilic strains *Coprothermobacter proteolyticus* and Methanothermobacter thermautotrophicus. J Biosci Bioeng.

[55] Zhao J, Westerholm M, Qiao W, Yin D, Bi S, Jiang M (2018). Impact of temperature and substrate concentration on degradation rates of acetate, propionate and hydrogen and their links to microbial community structure. Bioresour Technol.

[56] Maloney PC (1982). Energy coupling to ATP synthesis by the proton-translocating ATPase. J Membr Biol.

[57] Ragsdale Stephen W., Pierce Elizabeth (2008). Acetogenesis and the Wood–Ljungdahl pathway of CO2 fixation. Biochimica et Biophysica Acta (BBA) - Proteins and Proteomics.

[58] Zinder SH, Sowers KR, Ferry JG. *Methanosarcina thermophila* sp. nov., a thermophilic, Acetotrophic, methane-producing bacterium. Int J Syst Bacteriol. 1985;35:522–3.

[59] Dridi B, Fardeau ML, Ollivier B, Raoult D, Drancourt M (2012). *Methanomassiliicoccus luminyensis* gen. Nov., sp. nov., a methanogenic archaeon isolated from human faeces. Int J Syst Evol Microbiol.

