# Supplementary material for: Microbial activity response to hydrogen injection in thermophilic anaerobic digesters revealed by genome-centric metatranscriptomics
Source: Microbiome. 2018 Oct 27;6:194. doi: 10.1186/s40168-018-0583-4 (PMC6204281; doi:10.1186/s40168-018-0583-4)
Supplement: Supplementary file 1 — 16S rRNA gene amplicon results (Table S2 and Figures S1-S2), differentially expressed genes not assigned to MAGs (Figure S3), relative abundance of the 50 MAGs (Figure S4), statistical analysis (Figure S5), methanogenic pathways regulated by the discussed archaeal MAGs (Figures S5-S7), simulation results (Figures S9-S19) and mass balance calculations. (DOCX 5847 kb) [file 40168_2018_583_MOESM1_ESM.docx]

**Microbial activity response to hydrogen injection in thermophilic anaerobic digesters revealed by genome-centric metatranscriptomics**

Alessandra Fontana; Panagiotis G. Kougias; Laura Treu; Adam Kovalovszki; Giorgio Valle; Fabrizio Cappa; Lorenzo Morelli; Irini Angelidaki; Stefano Campanaro

**Additional file 1**

NGS data statistics and 16S rRNA gene amplicon results (Table S1 to S2 and Fig. S1 to S2)

Differentially expressed genes not assigned to MAGs (Fig. S3)

Relative abundance of the 50 MAGs (Fig. S4)

Statistical analysis (Fig. S5)

Methanogenic pathways regulated by the discussed archaeal MAGs (Fig. S6 to S8)

Simulation results (Figures S9 to S19)

Mass balance calculations

**Table S1** Statistics regarding raw and filtered data related to all NGS analyses obtained in the current study. Replicates samples for microbial analysis (a, b, c) have been collected in R1, R2 and R3 at three time-points in Phase I, meaning before H_2_ injection (I, II, III), and at one time point in Phase II, meaning after H_2_ injection (H). The total number of raw reads and the number of reads after pair-merging and filtering process have been reported per each replica for the three NGS methods applied in the present study: 16S rRNA gene amplicon sequencing (16S amplicons), total random metagenomics (TRS gDNA) and metatranscriptomics (RNA-seq).

|  | **16S amplicons** | | **TRS gDNA** | | **RNA-seq** | |
| --- | --- | --- | --- | --- | --- | --- |
| **ID** | **Raw reads** | **Merged and filtered** | **Raw reads** | **Merged and filtered** | **Raw reads** | **Merged and filtered** |
| **R1-I-a** | 256626 | 91200 | - | - | - | - |
| **R1-I-b** | 492408 | 139746 | - | - | - | - |
| **R1-I-c** | 362602 | 120683 | - | - | - | - |
| **R1-II-a** | 297924 | 89648 | - | - | - | - |
| **R1-II-b** | 275710 | 87090 | - | - | - | - |
| **R1-II-c** | 303426 | 104522 | - | - | - | - |
| **R1-III-a** | 268378 | 95562 | 10287776 | 8818995 | 20940340 | 20536432 |
| **R1-III-b** | 237382 | 83307 | 17192444 | 14768055 | 23052600 | 22613424 |
| **R1-III-c** | 220274 | 76683 | 11347582 | 9714979 | 18232671 | 17754036 |
| **R1-H-a** | - | - | 7804280 | 6587305 | 32812271 | 32203531 |
| **R1-H-b** | - | - | 8066664 | 6939638 | 30410352 | 29856461 |
| **R1-H-c** | - | - | 10235162 | 8662206 | 29543921 | 29013189 |
| **R2-III-a** | - | - | 7720826 | 6454683 | - | - |
| **R2-III-b** | - | - | 10219246 | 8561994 | - | - |
| **R2-III-c** | - | - | 9912878 | 8299553 | - | - |
| **R2-H-a** | - | - | 19631228 | 16360486 | - | - |
| **R2-H-b** | - | - | 18809546 | 15468619 | - | - |
| **R2-H-c** | - | - | 16585754 | 13716479 | - | - |
| **R3-I-a** | 601274 | 165286 | - | - | - | - |
| **R3-I-b** | 517310 | 150902 | - | - | - | - |
| **R3-I-c** | 288742 | 82693 | - | - | - | - |
| **R3-II-a** | 358664 | 115412 | - | - | - | - |
| **R3-II-b** | 357146 | 121341 | - | - | - | - |
| **R3-II-c** | 250220 | 83510 | - | - | - | - |
| **R3-III-a** | 345368 | 107050 | 12483892 | 10590424 | 30112439 | 29555098 |
| **R3-III-b** | 254714 | 73099 | 12757550 | 10848501 | 26909201 | 26429086 |
| **R3-III-c** | 453518 | 124249 | 11390552 | 9800708 | 32486849 | 31861174 |
| **R3-H-a** | - | - | 20172266 | 17496525 | 31533889 | 31006723 |
| **R3-H-b** | - | - | 11193188 | 9722547 | 29341280 | 28785157 |
| **R3-H-c** | - | - | 8862690 | 7641383 | 30806835 | 30240013 |

**Table S2** Microbial community analysis of samples collected in R1 and R3 at multiple time-points. A comparison between the microbial species putatively identified using the 16S rRNA gene amplicon sequencing and the dominant MAGs reconstructed using total random sequencing (TRS) is showed. OTUs were obtained at three time-points in Phase I (I, II, III), while MAGs are only related to the third time point (R1-TRS, R3-TRS). Relative abundances averages and standard deviations of the OTUs in R1 (R1-I, R1-II, R1-III) and R3 (R3-I, R3-II, R3-III) are reported. The OTUs’ values determined at the third time point were coherent with MAGs relative abundances averages estimated using coverage values.

| 16S rRNA amplicon OUTs | R1-I | R1-II | R1-III |  | R3-I | R3-II | R3-III |  | TRS MAGs | R1-TRS | R3-TRS |
| --- | --- | --- | --- | --- | --- | --- | --- | --- | --- | --- | --- |
| *D. tunisiensis* | 35.04% | 45.56% | 34.04% |  | 66.83% | 45.05% | 42.60% |  | ***D. tunisiensis* UC0050** | 17.32% | 27.78% |
| *C. proteolyticus* | 22.32% | 18.47% | 27.36% |  | 8.49% | 11.24% | 7.79% |  | ***C. proteolyticus* UC0011** | 43.14% | 10.63% |
| *A. hydrogeniformans* | 16.50% | 15.72% | 10.58% |  | 3.63% | 1.43% | 5.26% |  | ***A. hydrogeniformans* UC0046** | 17.10% | 7.71% |
| *Clostridiales* spp. | 19.10% | 15.76% | 23.80% |  | 0.29% | 19.34% | 17.04% |  | **Clostridiales sp. UC0015** | 8.59% | 6.55% |
| *A. mobile* | 1.86% | 0.79% | 1.14% |  | 13.27% | 5.98% | 3.33% |  | ***A. mobile* UC0048** | 1.65% | 4.68% |
| *T. acetatoxydans* | 2.46% | 1.36% | 1.44% |  | 1.95% | 0.52% | 0.01% |  | ***T. acetatoxydans* UC0018** | 4.28% | 0.06% |
| *Bacteroidales* spp. | 0.72% | 0.44% | 0.20% |  | 1.94% | 10.92% | 16.05% |  | ***Bacteroidetes* sp. UC0002** | 0.01% | 10.16% |
| *Syntrophomona*s sp. | 0.02% | 0.02% | 0.01% |  | 0.38% | 0.98% | 1.61% |  | ***Syntrophomonas* sp. UC0014** | 0.10% | 4.87% |
| *Anaerolineaceae* sp. | 0.00% | 0.01% | 0.00% |  | 0.03% | 0.55% | 1.45% |  | ***Chloroflexi* sp. UC0004** | 0.01% | 2.61% |
| *S. schinkii* | 0.00% | 0.00% | 0.00% |  | 0.25% | 0.17% | 1.12% |  | ***Syntrophaceticus* sp. UC0017** | 0.06% | 3.65% |
| *Haloplasmataceae* sp. | 0.00% | 0.00% | 0.00% |  | 0.01% | 0.81% | 0.72% |  | ***Firmicutes* sp. UC0013** | 0.02% | 1.91% |
| *Anaerobaculum* sp. | 0.48% | 0.81% | 0.43% |  | 0.30% | 0.05% | 0.31% |  | ***Anaerobaculum* sp. UC0047** | 0.59% | 1.96% |
| *Methanothermobacter* sp. | 0.36% | 0.19% | 0.33% |  | 0.69% | 0.09% | 0.22% |  | ***M. wolfeii* UC0008** | 1.09% | 1.02% |
| *M. thermophila* | 0.00% | 0.00% | 0.00% |  | 0.09% | 0.02% | 0.06% |  | ***M. thermophila* UC0006** | 0.04% | 0.66% |

Several OTUs having variable relative abundances between different time-points in R3 were could not be classified at high taxonomical level. In particular, 4 OTUs were grouped as *Clostridiales* spp. and 2 OTUs were assigned to *Bacteroidales* spp. A possible explanation of these differences may be related to the limitation of 16S rRNA gene amplicon technique; in fact, information obtained from such a small fragment of DNA are not enough to discriminate the specific microbes that may be responsible for the anaerobic digestion process. Moreover, the major variations between time points were recorded in OTUs assigned to *Tepidanaerobacter* *acetatoxydans*, *Haloplasmataceae* sp. and *Anaerolineaceae* sp. (35-fold, 18-fold and 13-fold changes on average, respectively). These dynamic OTUs showed two opposite trends: *T. acetatoxydans* was clearly disappearing with time, while *Haloplasmataceae* sp. and *Anaerolineaceae* sp. were increasing in abundance. This finding indicates a potential functional redundancy in this particular AD system, meaning that the two OTUs presenting increased growth could substitute the role of *T. acetatoxydans* in the microbial consortium. Indeed, *Haloplasmataceae* and *Anaerolineaceae* families are known to include fermenting bacteria species which were found to be positively correlated to acetate concentrations in anaerobic digesters [1,2]. Therefore, it could be possible that these two microbes functionally replaced *T. acetatoxydans* in both reactor configurations, during the progressive increment of acetate levels. It can be assumed that such conditions created a more suitable environment for these two species, resulting in a faster growth.

**References**1. Li YF, Nelson MC, Chen PH, Graf J, Li Y, Yu Z. Comparison of the microbial communities in solid-state anaerobic digestion (SS-AD) reactors operated at mesophilic and thermophilic temperatures. Appl. Microbiol. Biotechnol. 2014;

2. Yi J, Dong B, Jin J, Dai X. Effect of increasing total solids contents on anaerobic digestion of food waste under mesophilic conditions: Performance and microbial characteristics analysis. PLoS One. 2014


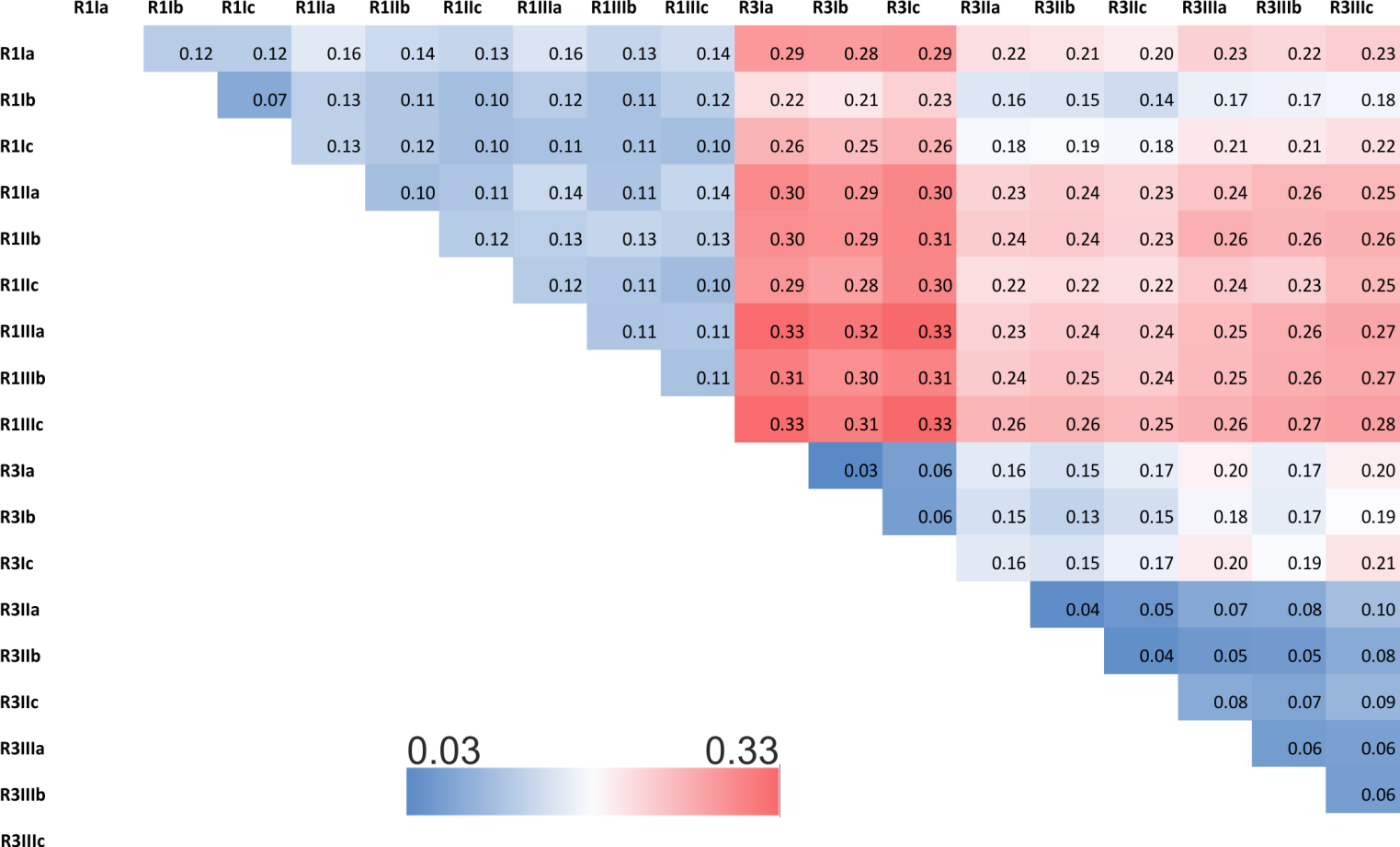


**Fig. S1** β-diversity calculated among the samples. Results obtained for β-diversity calculation (Whittaker method) were visualized as a heatmap. Correspondence between colors and β-diversity values is reported in the scale at the bottom of the matrix. Average β-diversity values were 0.12 for samples collected in R1, 0.12 for samples in R3 and 0.25 for comparisons between R1 and R3.


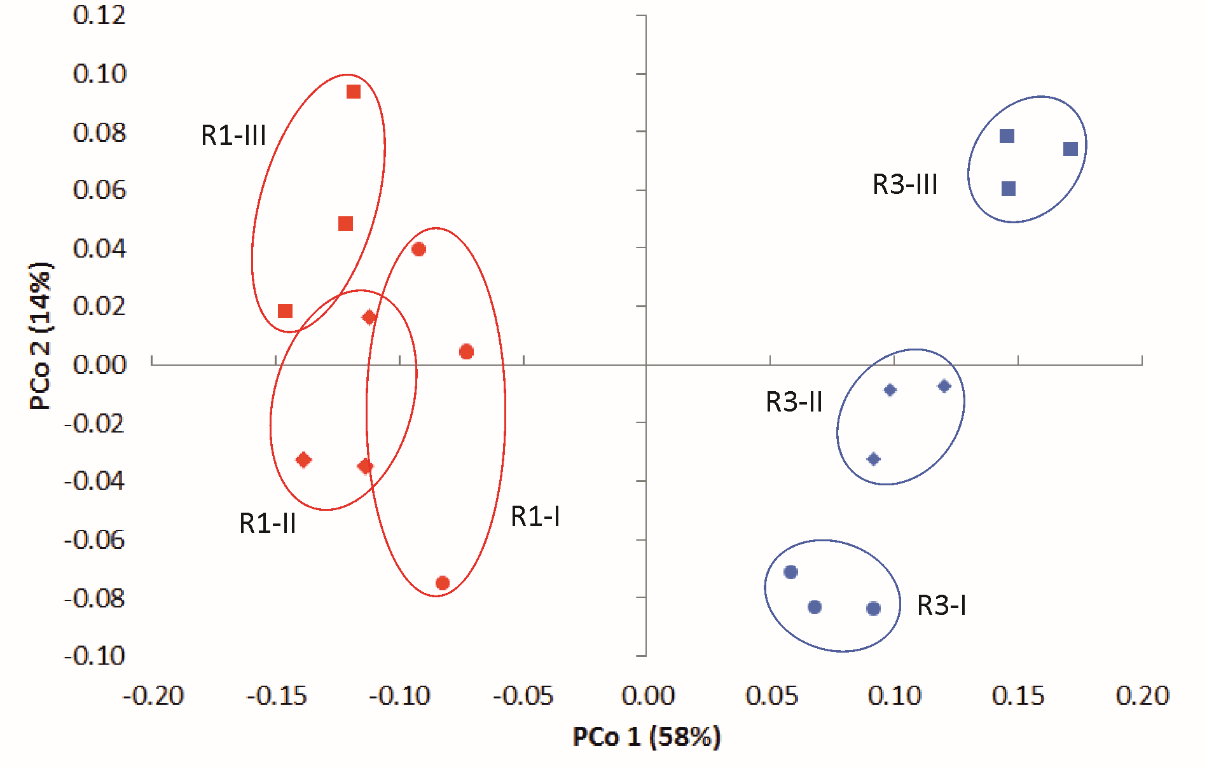


**Fig. S2** Principal coordinate analysis (PCoA) plot representing variations of the OTUs based on phylogenetic distance and Unweighted UniFrac method. Replicate samples obtained from the two reactors (R1 and R3) at three time-points (I, II and III) are showed as uncorrelated linear principal coordinates. The two coordinates represented in the graph contribute to more than 70% of total variance (PCo1 58% and PCo2 14%).

**
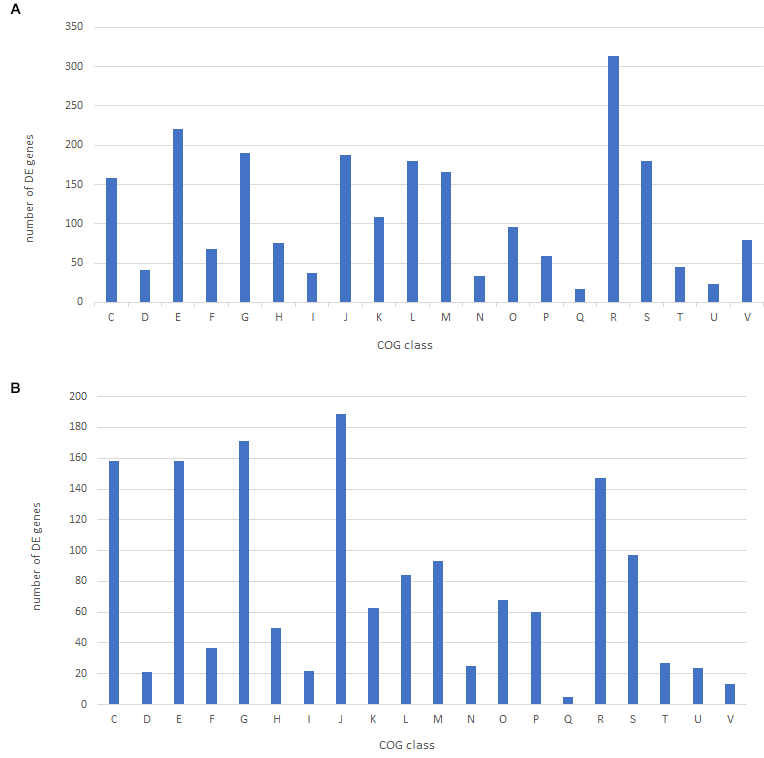
**

**Fig. S3** Number of total differentially expressed genes (not assigned to MAGs) per each COG category, in the single stage (A) and two-stage (B) reactors.

**
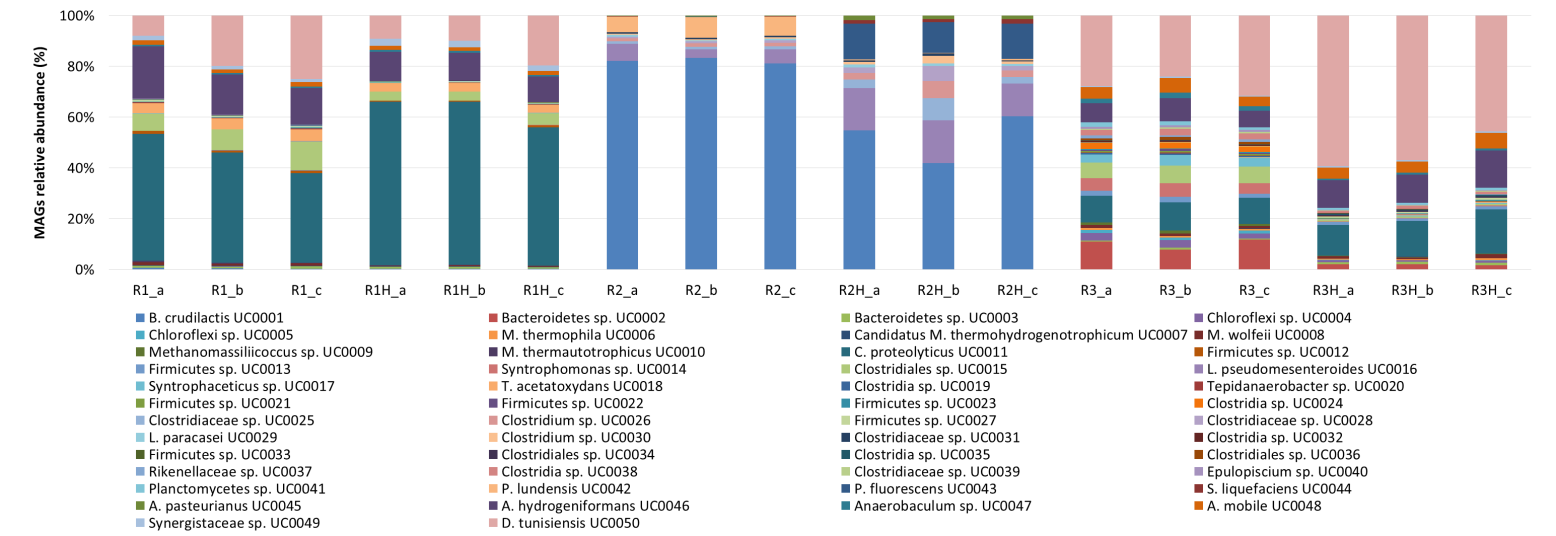
**

**Fig. S4** Relative abundance of the 50 MAGs distributed in the different reactor configurations. R1 and R1H: single stage pre- and post-H2, respectively; R2 and R2H: acidogenic reactor of the two-stage pre- and post-H2, respectively; R3 and R3H: methanogenic reactor of the two-stage pre- and post-H2, respectively; a, b, c: replicates.

**
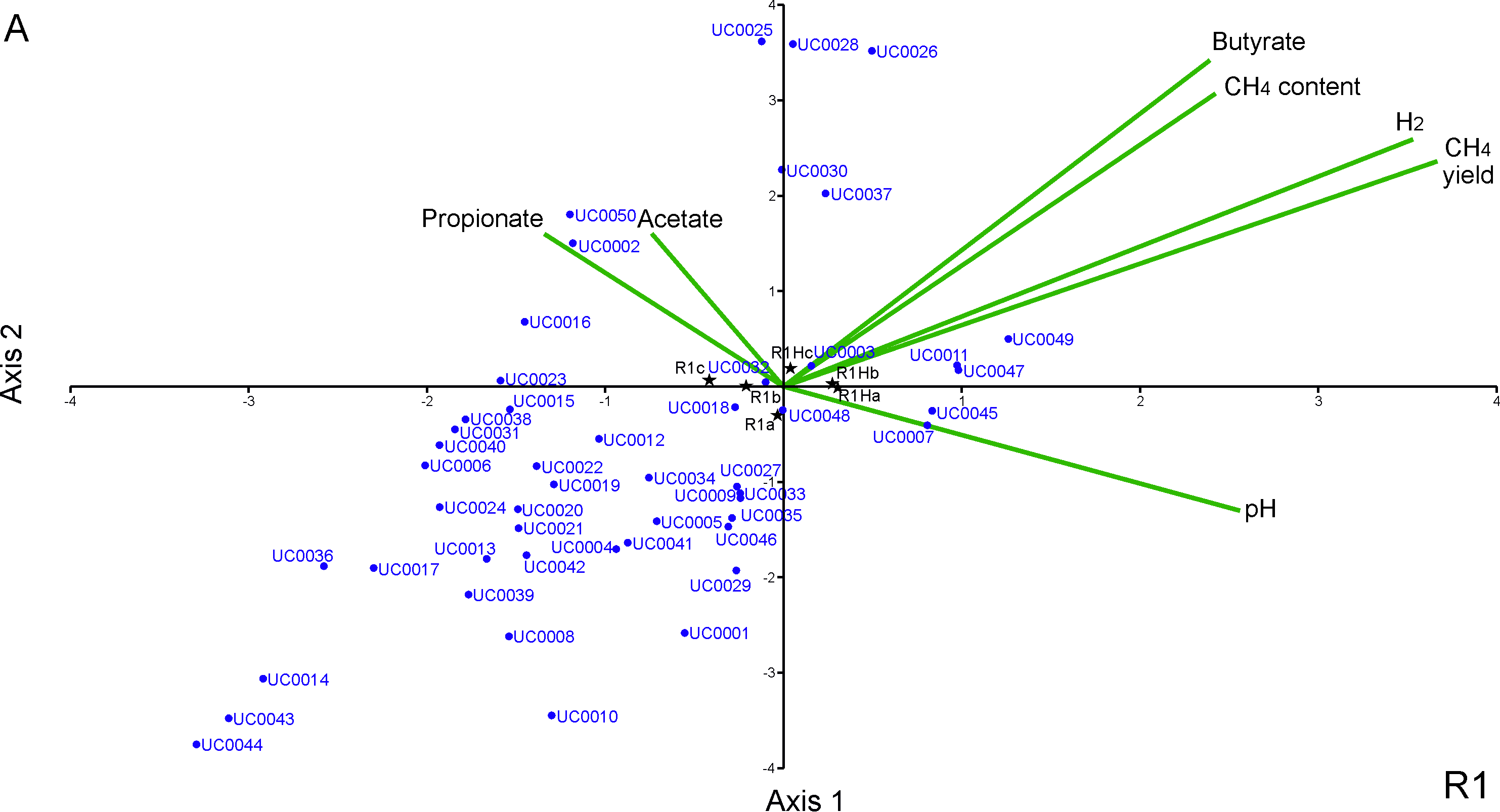
**

**
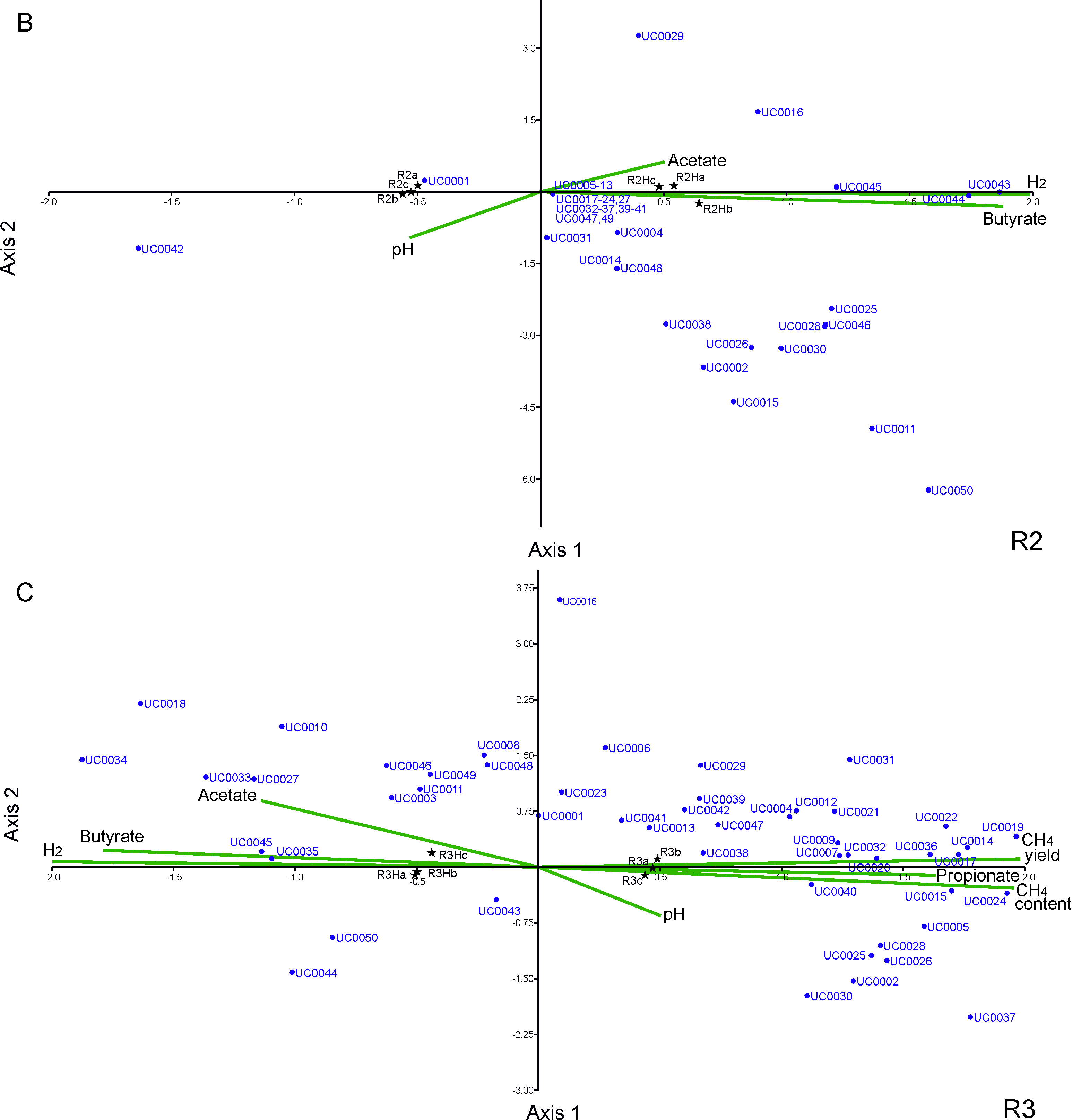
**

**Fig. S5** Canonical correspondence analysis (CCA) testing significative correlations between relative abundance of the MAGs and reactor parameters, in the single stage reactor (A), acidogenic reactor of the two-stage configuration (B), and methanogenic reactor of the two-stage configuration (C). R1 and R1H: single stage pre- and post-H_2_, respectively; R2 and R2H: acidogenic reactor of the two-stage pre- and post-H_2_, respectively; R3 and R3H: methanogenic reactor of the two-stage pre- and post-H_2_, respectively; a, b, c: replicates).

**
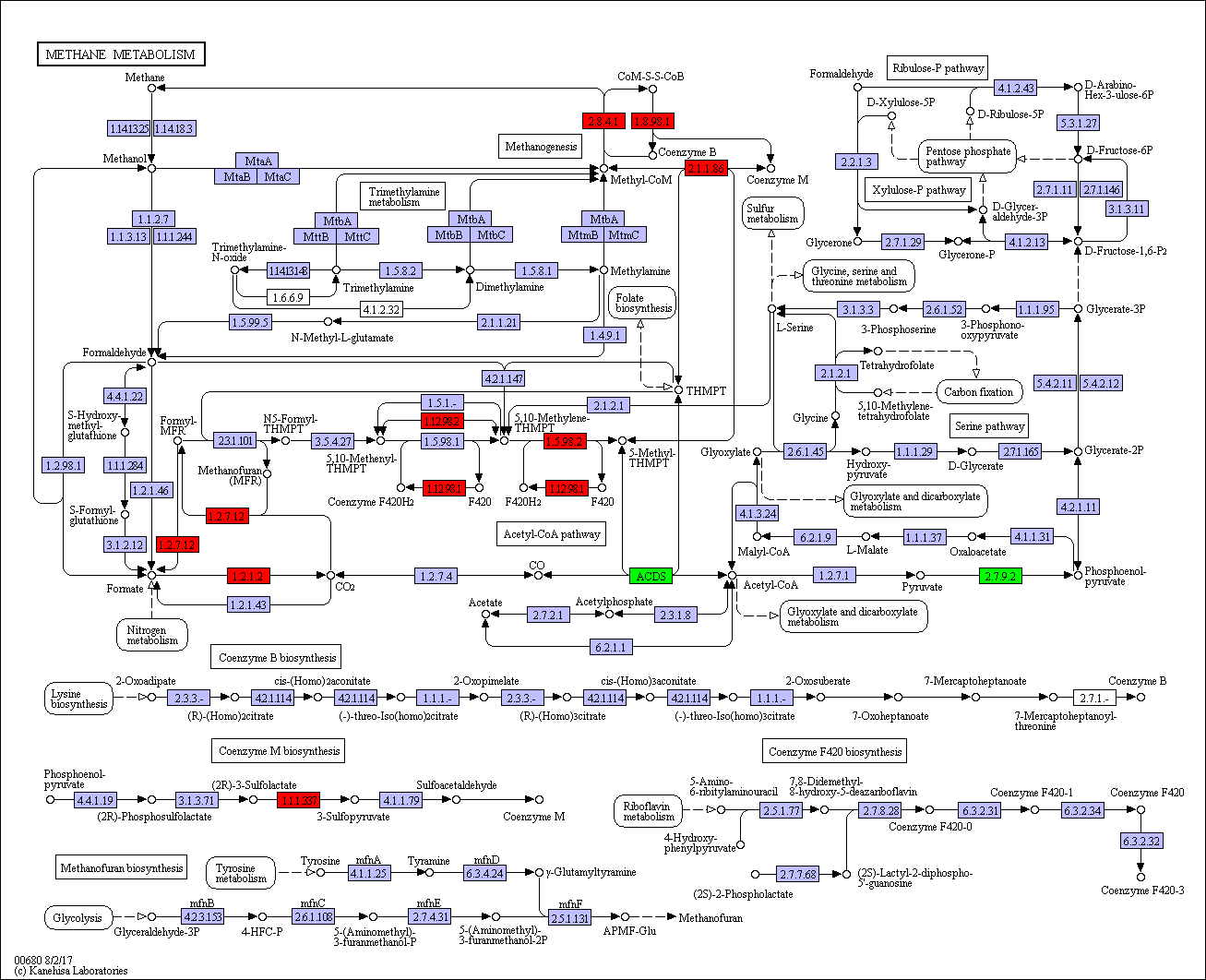
**

**Fig. S6** KEGG methane metabolism of *Methanothermobacter wolfeii* UC0008 in the single stage reactor (R1). Over and under expressed genes are labeled in red and green, respectively.


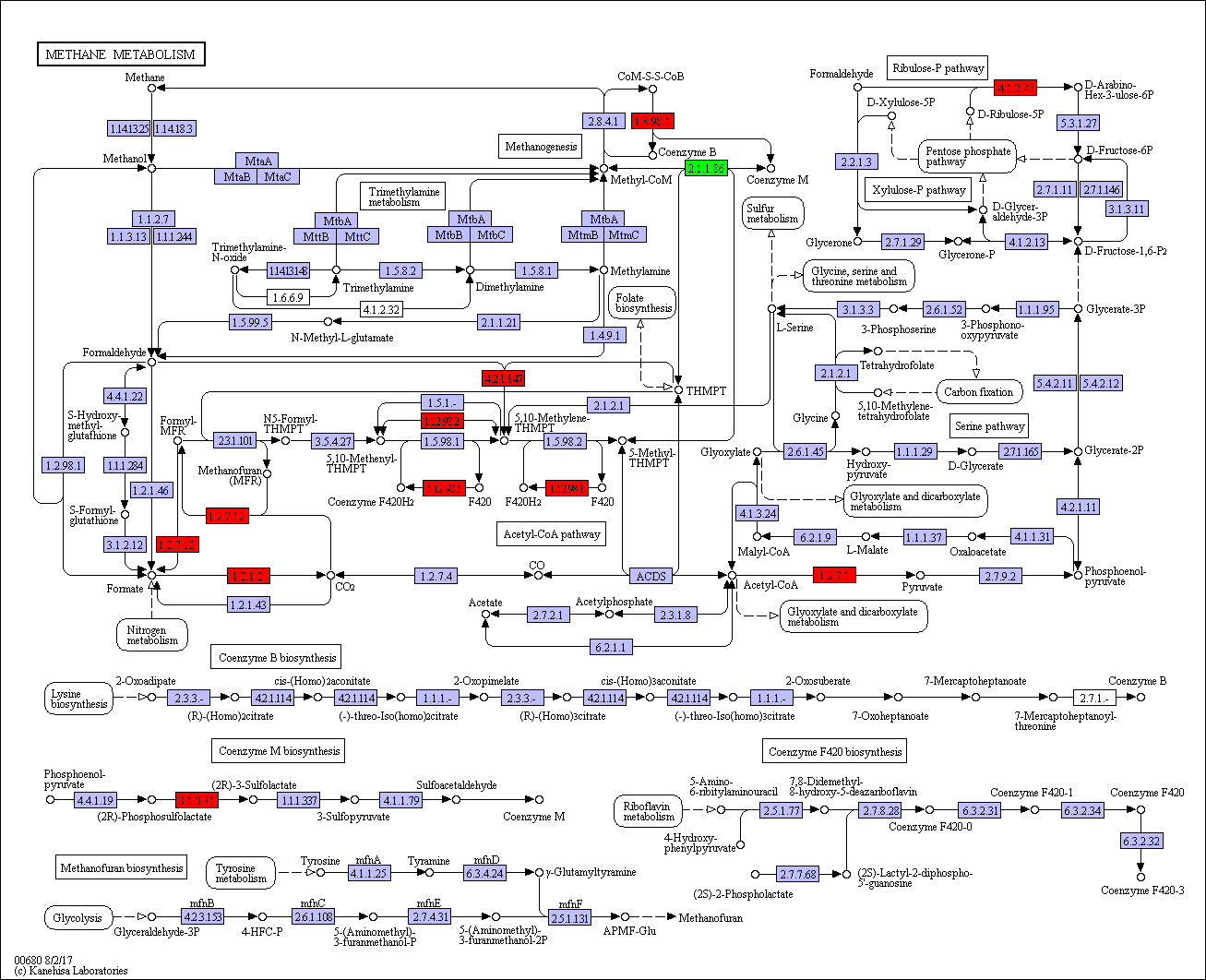


**Fig. S7** KEGG methane metabolism of *Methanothermobacter wolfeii* UC0008 in the methanogenic reactor of the serial configuration (R3). Over and under expressed genes are labeled in red and green, respectively.


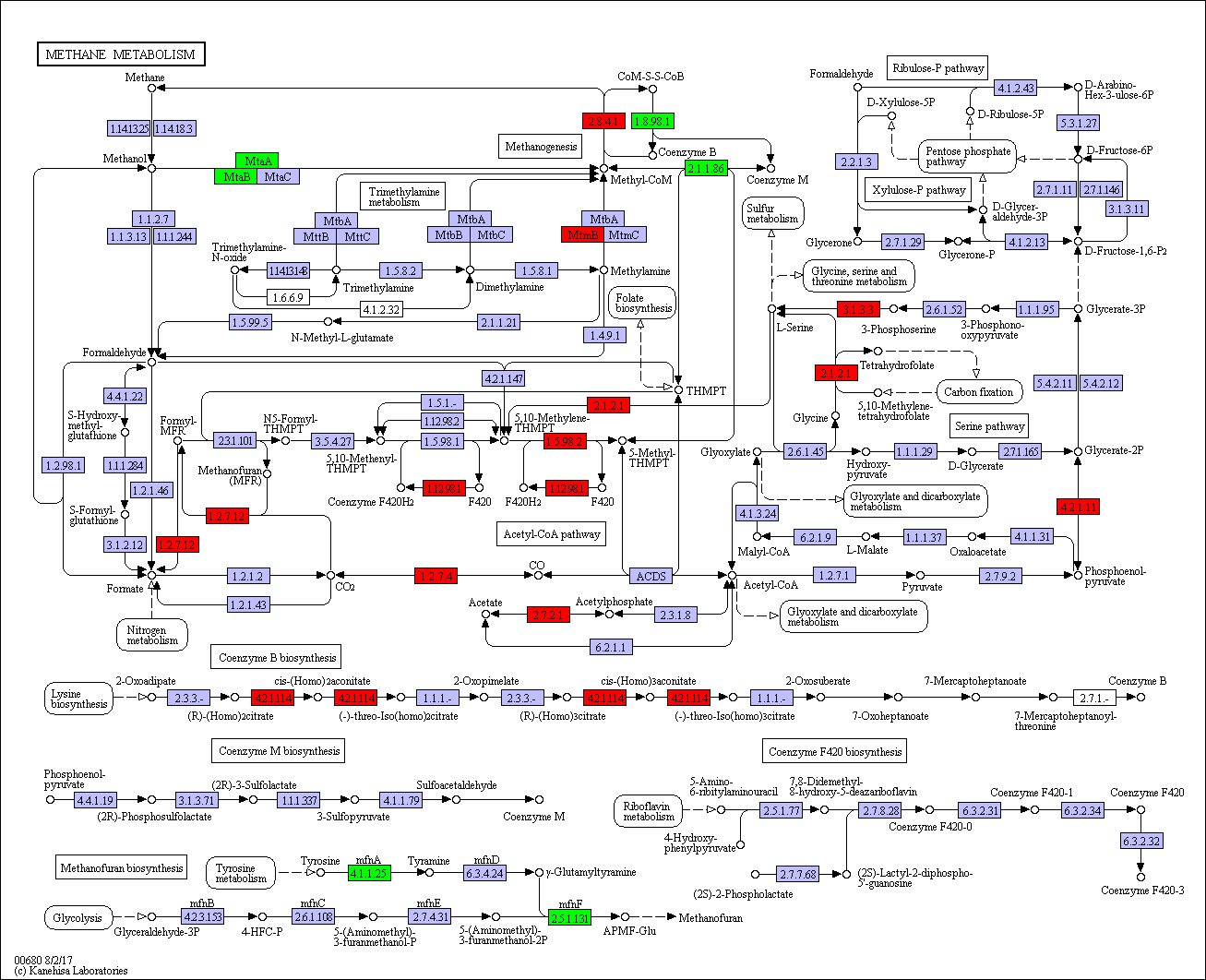


**Fig. S8** KEGG methane metabolism of *Methanosarcina thermophila* UC006 in the methanogenic reactor of the serial configuration (R3). Over and under expressed genes are labeled in red and green, respectively.

**SIMULATION RESULTS**

**Single stage reactor:**


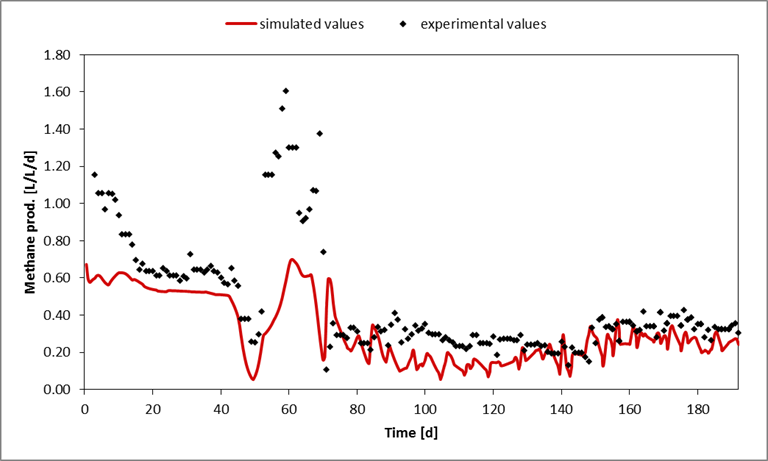


**Fig. S9** Methane productivity in R1.

**
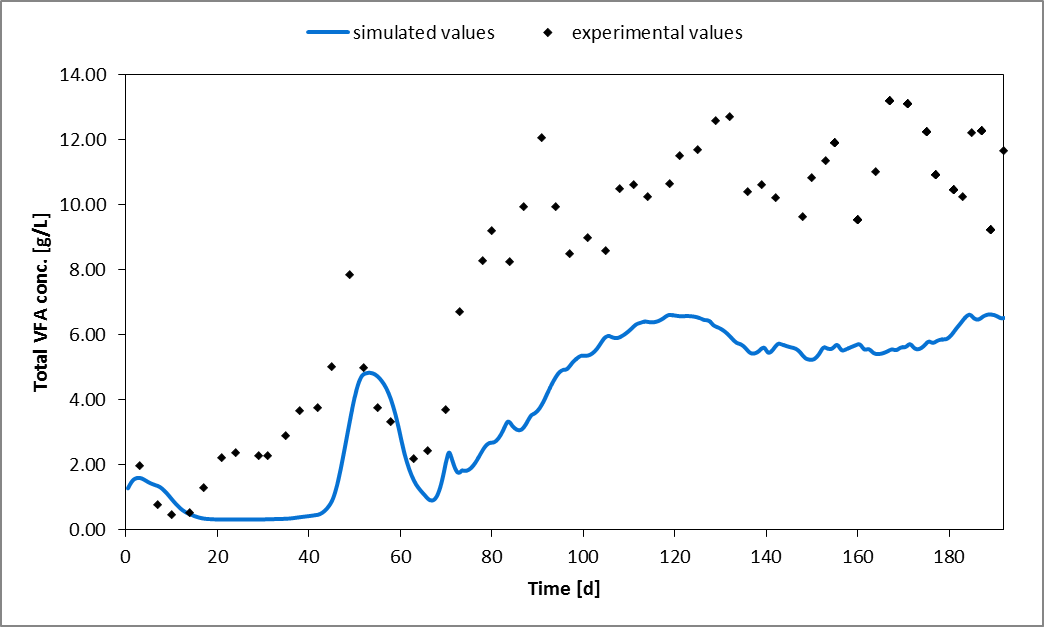
**

**Fig. S10** Total VFA concentration in the digestate of R1.

**
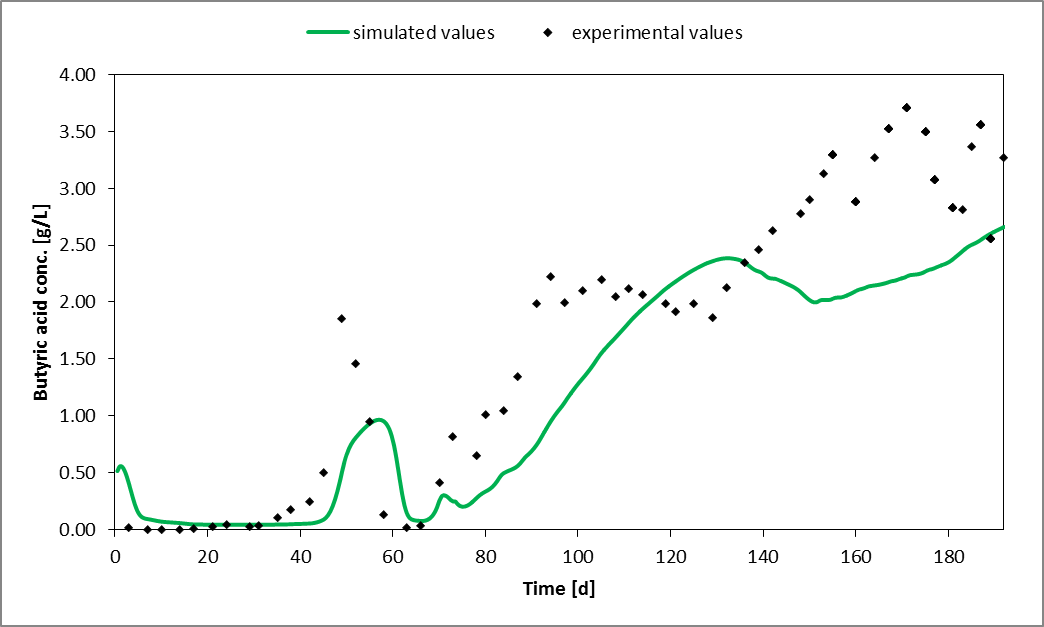
**

**Fig. S11** Butyric acid concentration in the digestate of R1.


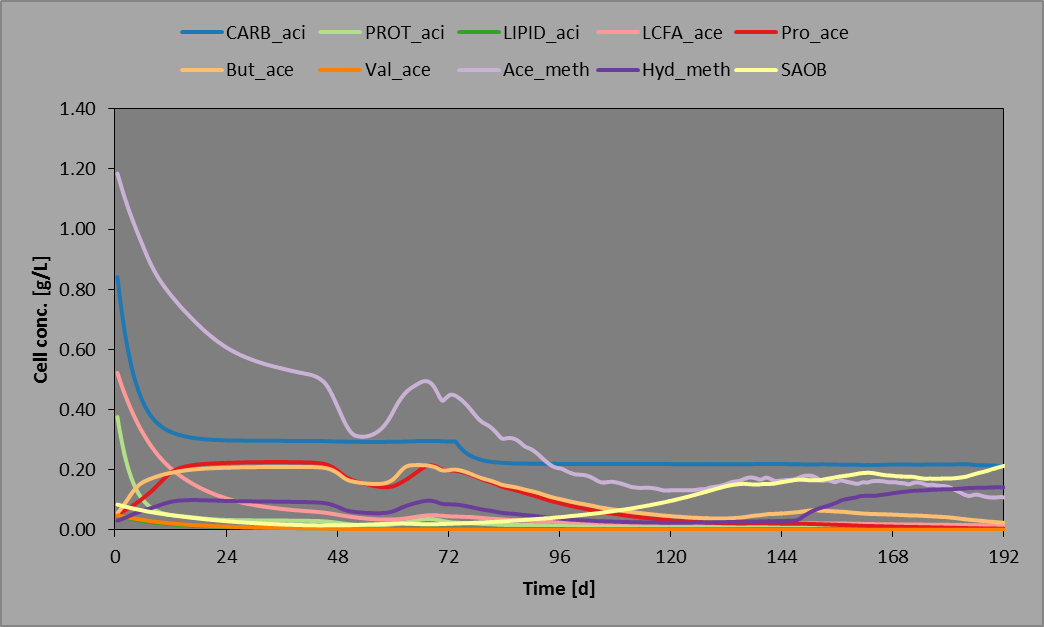


**Fig. S12** Concentration of different simulated microbial groups in R1. *CARB_aci*, *PROT_aci* and *LIPID_aci* are acidogenic bacterial groups; *LCFA_ace*, *Pro_ace*, *But_ace* and *Val_ace* are acetogenic bacterial groups; *Ace_meth* and *Hyd_meth* are methanogenic archaeal groups; *SAOB* are syntrophic acetate oxidixing bacterial groups. Initial concentrations were defined by fitting the initial experimental data points of other variables to their respective simulation curves. The results are not quantitative, providing only limited information about the succession dynamics of the microbial community.

**Two-stage, acidogenic reactor:**

**
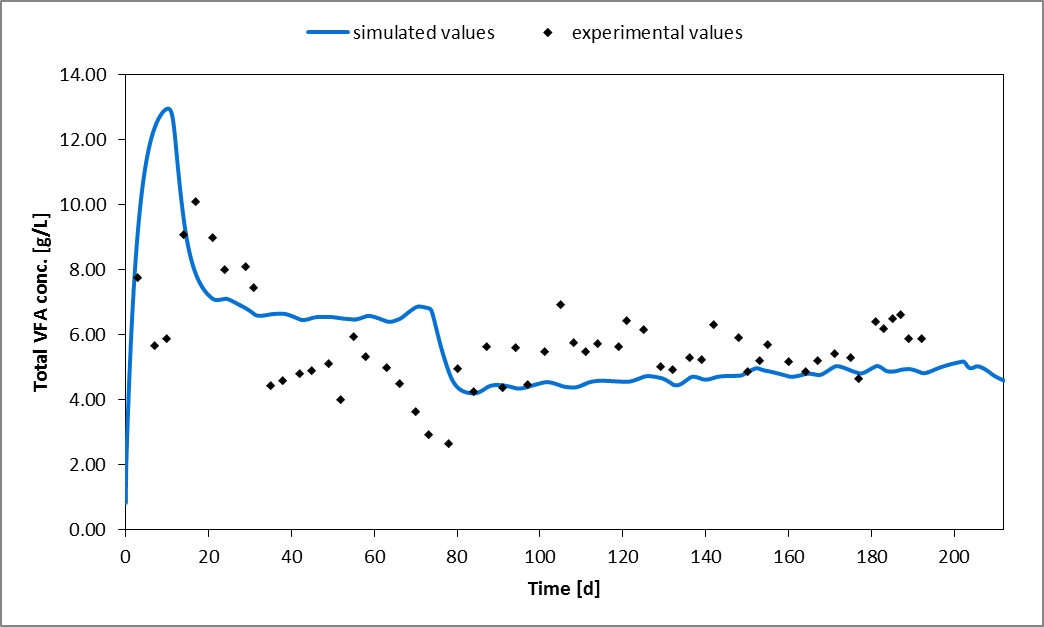
**

**Fig. S13** Total VFA concentration in the digestate of R2.


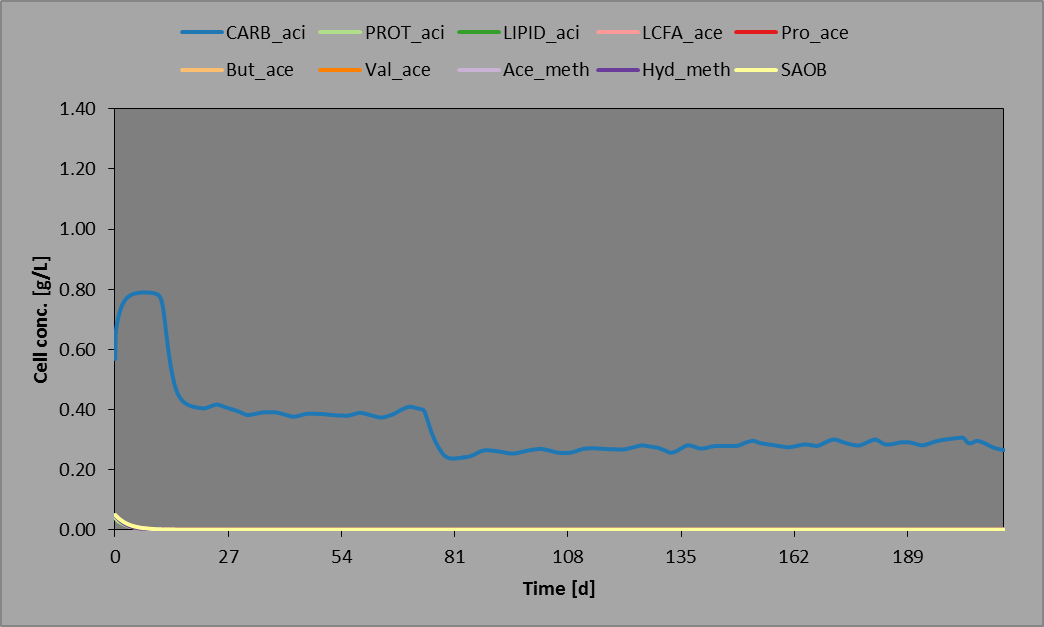


**Fig. S14** Concentration of different simulated microbial groups in R2. *CARB_aci*, *PROT_aci* and *LIPID_aci* are acidogenic bacterial groups; *LCFA_ace*, *Pro_ace*, *But_ace* and *Val_ace* are acetogenic bacterial groups; *Ace_meth* and *Hyd_meth* are methanogenic archaeal groups; *SAOB* are syntrophic acetate oxidixing bacterial groups. Initial concentrations were defined by fitting the initial experimental data points of other variables to their respective simulation curves. The results are not quantitative, providing only limited information about the succession dynamics of the microbial community.

**Two-stage, methanogenic reactor:**

**
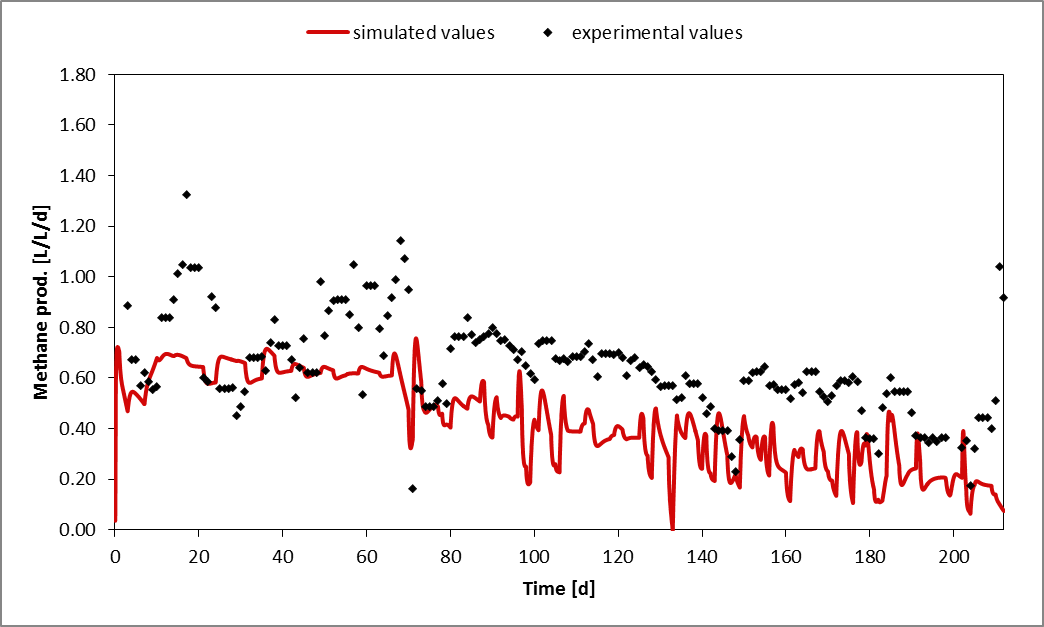
**

**Fig. S15** Methane productivity in R3.

**
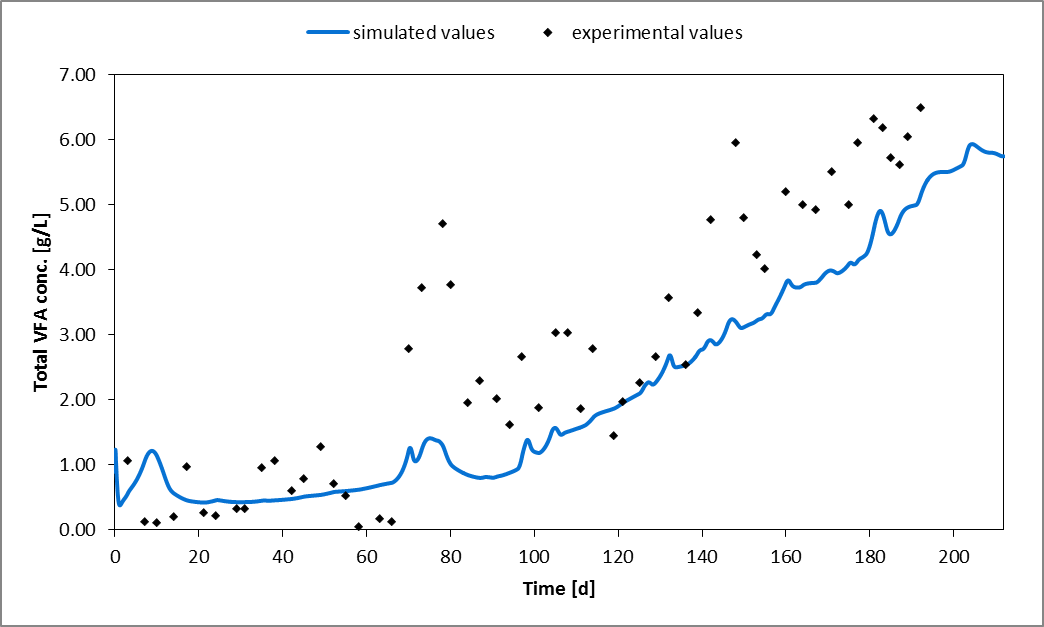
**

**Fig. S16** Total VFA concentration in the digestate of R3.

**
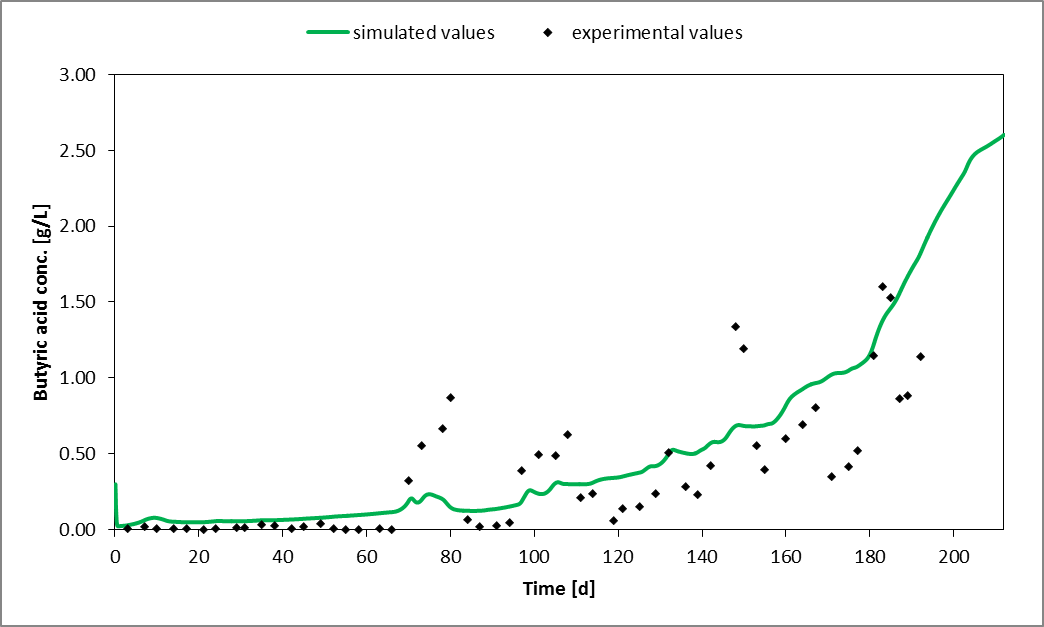
**

**Fig. S17** Butyric acid concentration in the digestate of R3.

**
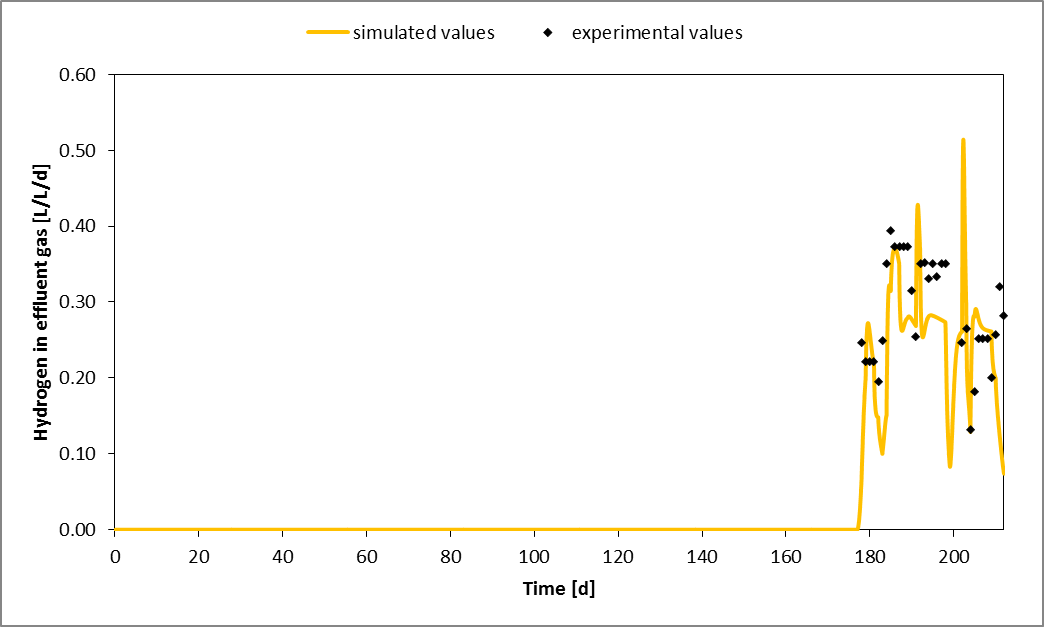
**

**Fig. S18** Hydrogen fraction in the effluent gas of R3.

**
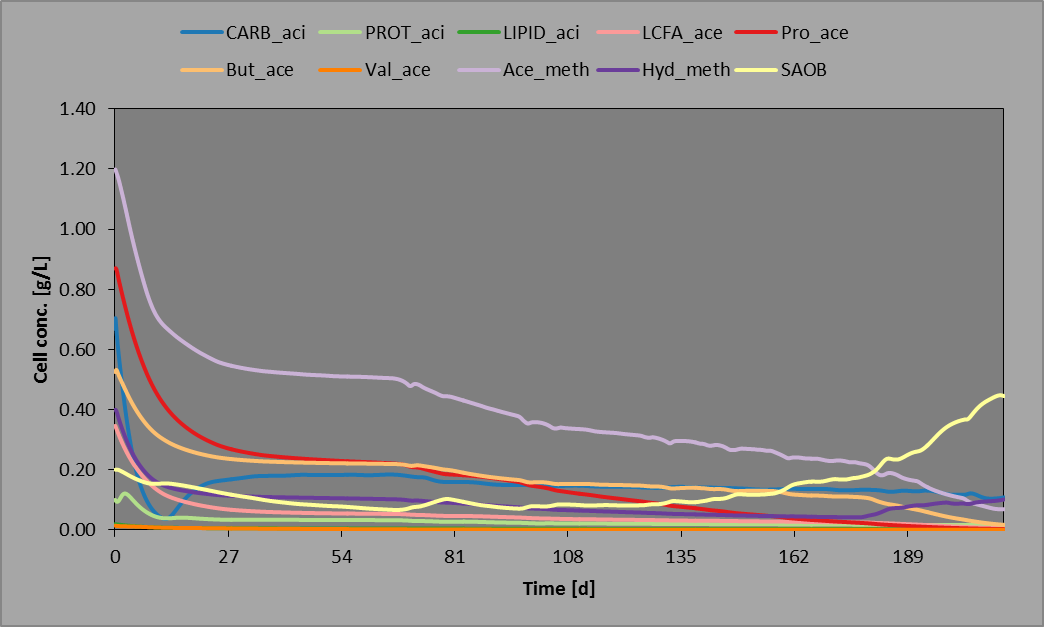
**

**Fig. S19** Concentration of different simulated microbial groups in R3. *CARB_aci*, *PROT_aci* and *LIPID_aci* are acidogenic bacterial groups; *LCFA_ace*, *Pro_ace*, *But_ace* and *Val_ace* are acetogenic bacterial groups; *Ace_meth* and *Hyd_meth* are methanogenic archaeal groups; *SAOB* are syntrophic acetate oxidixing bacterial groups. Initial concentrations were defined by fitting the initial experimental data points of other variables to their respective simulation curves. The results are not quantitative, providing only limited information about the succession dynamics of the microbial community.

**MASS BALANCE CALCULATIONS**

Reactions considered:

Acetogenesis $2{CO}_{2}+4H_{2}\to{CH}_{3}COOH+2H_{2}O$

Propionate formation ${CH}_{3}COOH+3H_{2}+{CO}_{2}\to{CH}_{3}{CH}_{2}COOH+2H_{2}O$

Butyrate formation ${CH}_{3}{CH}_{2}COOH+3H_{2}+{CO}_{2}\to{CH}_{3}{CH}_{2}{CH}_{2}COOH+2H_{2}O$

Hydrogenotrophic methanogenesis $4H_{2}+{CO}_{2}\to{CH}_{4}+2H_{2}O$

moles_H2, injected /day_ = V_H2, injected /day_ /V_molar, ideal gas, 55°C_ = 2.45 L/day / 26.9 L/mol= 0.0911 mol/day

**Single stage reactor:**

- Methane increase (Δmethane) after H_2_= 0.24 L/day

moles_Δmethane_= 0.24 L/day / 26.9 L/mol = 0.0089 mol

moles_H2, used for Δmethane_ = 0.0089 x 4 = 0.0357 mol (~40% of the H_2_ moles injected is utilized for the methane increase)

- Butyrate increase (Δbutyrate) after H_2_= 1.2 g/L

moles_Δbutyrate_= 1.2 g/L x 3 Lr = 3.6 g / 88.1 g/mol = 0.04086 mol

Hypothesis a): moles_H2, used for Δbutyrate (from CO2)_ = 0.04086 mol x 10 mol H_2_ = 0.409 mol

Hypothesis b): moles_H2, used for Δbutyrate (from acetate)_ = 0.04086 mol x 6 mol H_2_ = 0.245 mol

Hypothesis c): moles_H2, used for Δbutyrate (from propionate)_ = 0.04086 mol x 3 mol H_2_ = 0.123 mol

The left 60% H_2_ moles injected (0.0554 mol) are not enough for the butyrate increment registered, considering all hypotheses (a, b and c). Thus, the utilization of indigenous H_2_ should be considered:

moles_H2, BIOGAS_ = 0.48 L/day / 26.9 L/mol = 0.018 mol

moles_H2, left for other metabolisms_ = 0.055 – 0.018 = 0.037 mol

Hypothesis a): 0.409-0.037 = 0.37 mol (too much indigenous H_2_ required to produce butyrate from CO_2_, not reasonable)

Hypothesis b): 0.245-0.037 = 0.21 mol (too much indigenous H_2_ required to produce butyrate from acetate, not reasonable)

Hypothesis c): 0.123-0.037 = 0.09 mol (most suitable hypothesis)

Considering hypothesis c) and the equation for lactose fermentation to acetate and butyrate (main VFA in R1):

$$C_{12}H_{22}O_{11}+5H_{2}O \to8H_{2}+4{CO}_{2}+4{CH}_{3}COOH$$

$$C_{12}H_{22}O_{11}+H_{2}O \to4H_{2}+4{CO}_{2}+2{CH}_{3}{CH}_{2}{CH}_{2}COOH$$

moles lactose in the feed/day = 5.6 g/ 342.3 g/mol = 0.0164 mol

moles H_2_ from lactose fermentation to acetate = 0.0164 x 8=0.13 mol

moles H_2_ from lactose fermentation to butyrate = 0.0164 x 4=0.07 mol

It is therefore possible to consider the utilization of indigenous hydrogen to explain the butyrate increment via propionate reduction (this last VFA is indeed the lowest in concentration).

**Two-stage, acidogenic reactor:**

- Butyrate increase (Δbutyrate) after H_2_= 1.3 g/L

moles_Δbutyrate_= 1.3 g/L x 0.6 Lr = 0.78 g / 88.1 g/mol = 0.0089 mol

Hypothesis a): moles_H2, used for Δbutyrate_ _(from CO2)_ = 0.0089 mol x 10 mol H_2_ = 0.089 mol (~97% of the H_2_ injected)

Hypothesis b): moles_H2, used for Δbutyrate (from acetate)_ = 0.0089 mol x 6 mol H_2_ = 0.053 mol (~60% of the H_2_ injected)

Propionate reduction hypothesis has not been considered since propionate concentration was negligible in this reactor (0.020 ± 0.003 g/L).

**Two-stage, methanogenic reactor:**

moles_H2, BIOGAS_ = 0.94 L/day / 26.9 L/mol = 0.035 mol

Hypothesis a): moles _H2, from R2_ = (0.091 – 0.089) mol = 0.003 mol (no injected H_2_ goes to R3)

Hypothesis b): moles _H2, from R2_ = (0.091 – 0.053) mol = 0.038 mol (~equal to the H_2_ moles in the output biogas; thus, probably not utilized for other metabolic reactions)

Thus, the H_2_ injected in R2 did not reach R3 (hypothesis a) or was not utilized (hypothesis b). Since the concentration of acetate slightly decreased in R2, and 30% of the injected hydrogen was found in the effluent gas of R3, hypothesis b) seems the most suitable.

- Butyrate increase (Δbutyrate) after H_2_= 0.7 g/L (most probably from R2 and/or acetogenesis inhibition in R3, given acetate concentration)

moles_Δbutyrate_= 0.7 g/L x 2.4 Lr = 1.68 g / 88.1 g/mol = 0.0191 mol

- Acetate increase (Δacetate) after H_2_= 1.7 g/L

moles_Δacetate_= 1.7 g/L x 2.4 Lr = 4.08 g / 60.05 g/mol = 0.068 mol

Most of these acetate moles probably derived from butyrate degradation:

$${CH}_{3}{CH}_{2}{CH}_{2}COOH+2H_{2}O\to2{CH}_{3}COOH+2H_{2}$$

moles_Δacetate from butyrate oxidation_ = 0.0191 mol_Δbutyrate_ x 2= 0.038 (~60% of the acetate increment experienced)

Acetogenesis account for acetate increase:

moles_Δacetate from acetogenesis_ = 0.029 mol/4 = 0.007 mol (~10% of the acetate increment experienced)

Thus, the left 30% of augmented acetate moles was probably due to an accumulation effect, which may have also inhibited the acetogenic pathway.
